# A Unified Framework for Association Analysis with Multiple Related Phenotypes

**DOI:** 10.1371/journal.pone.0065245

**Published:** 2013-07-05

**Authors:** Matthew Stephens

**Affiliations:** Department of Statistics and Department of Human Genetics, University of Chicago, Chicago, Illinois, United States of America; Queen's University Belfast, United Kingdom

## Abstract

We consider the problem of assessing associations between multiple related outcome variables, and a single explanatory variable of interest. This problem arises in many settings, including genetic association studies, where the explanatory variable is genotype at a genetic variant. We outline a framework for conducting this type of analysis, based on Bayesian model comparison and model averaging for multivariate regressions. This framework unifies several common approaches to this problem, and includes both standard univariate and standard multivariate association tests as special cases. The framework also unifies the problems of *testing* for associations and *explaining* associations – that is, identifying which outcome variables are associated with genotype. This provides an alternative to the usual, but conceptually unsatisfying, approach of resorting to univariate tests when explaining and interpreting significant multivariate findings. The method is computationally tractable genome-wide for modest numbers of phenotypes (e.g. 5–10), and can be applied to summary data, without access to raw genotype and phenotype data. We illustrate the methods on both simulated examples, and to a genome-wide association study of blood lipid traits where we identify 18 potential novel genetic associations that were not identified by univariate analyses of the same data.

## Introduction

The problem of assessing associations among multiple variables arises in a wide range of settings. Here we are motivated primarily by genetic association studies, which aim to assess associations between genetic variants and one or more phenotypes (observable characteristics) of interest, such as health-related quantitative traits (e.g. LDL-cholesterol, HDL-cholesterol) or disease status. However, many of the issues that arise in this setting also occur elsewhere, and so the statistical framework and results given here have potential for wider application.

In genome-wide association studies, published analyses are almost always univariate, considering each phenotype independently, even when multiple phenotypes are available on each individual (e.g. [1], to give just one example). However, in a sign that this may change in the future, the last few years have seen a plethora of papers related to multivariate association testing, including for example [2]–[10]; see also review papers by [11], [12]. Nonetheless, statistical methods for assessing associations with multiple traits remain surprisingly under-developed, and still more under-utilized.

The under-utilization of multivariate association methods may partly reflect a lack of general appreciation for the potential increased power of multivariate analyses. This is despite the fact that comparisons of multivariate and univariate association methods usually conclude that multivariate approaches can increase power. However, a more important factor may be that, despite their power, multivariate association analyses can be difficult to interpret. For example, rejecting a null hypothesis of no association does not indicate *which* phenotypes are associated, which is often the question of primary interest. In addition, some existing multivariate approaches for genetic data, while sophisticated, are also somewhat complex, which may discourage potential users.

Here we focus on relatively simple multivariate association analyses, involving a single genetic variant and a modest number of phenotypes (e.g. up to 10). Our aims include not only emphasizing the benefits of multivariate association analyses, but particularly to understand *when* and *why* a multivariate analysis will be most helpful, and, perhaps most importantly, to draw some connections between apparently disparate approaches. In particular we outline an analysis framework, based on model comparison, which effectively includes both standard univariate and standard multivariate association tests, as well as a large number of other standard tests, as special cases. Framing the association analysis as a model comparison problem, rather than as a testing problem focussed only on rejecting the null hypothesis, helps illuminate the settings under which each analysis approach will outperform others. It also provides an integrated way to both *test* for association and *interpret* associations, and in particular to address the primary question of which phenotypes are associated with each genetic variant.

The next section (Methods) provides i) further background and motivation; ii) a description of the framework in general terms; iii) detailed consideration of methods for the special case where a multivariate normal distribution can be used for the phenotypes; and iv) a discussion of challenges that may arise in practice when applying these methods. The methods for multivariate normal phenotypes are easily implemented (e.g. in R), and can be applied genome-wide, requiring only summary data, rather than individual genotype data (which can be harder to arrange access to, particularly when coordinating across multiple studies of the same phenotypes). In Results, we illustrate the methods on both simulated data and on a large meta-analysis of lipid-related traits, identifying several novel putative associations. The Discussion outlines connections between our framework and other work (particularly graphical models), highlights some of the main limitations and weaknesses, and suggests directions for future work.

## Methods

### Background and motivation

To illustrate some key issues, consider the following simple example. Suppose we have measured both *height* and *weight* on a random sample of (unrelated) genotyped individuals, and we wish to identify genetic variants that are associated with one or both of these phenotypes. In addition, having identified such variants, we wish to assess, for each one, *how* it is associated with the phenotypes. For example we would like to know whether it is associated with just *height*, just *weight*, or both. We refer to the first of these problems as *testing* for associations, and the second as *interpreting* the associations.

For simplicity, here and throughout this paper, we consider testing and interpreting associations with a *single* genetic variant 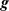, with the idea that any such analysis strategy would be applied to each measured genetic variant, one at a time. This is the approach taken by almost all GWAS analyses, although there can be advantages to analyzing multiple variants jointly: e.g. see [13], [14].

Even for a single genetic variant, and just two phenotypes, there are several simple association tests one might consider. These include:

1. Separate (univariate) tests for association with each of *weight* and *height*.
2. A test for association with *weight controlling for height*. (This analysis is roughly equivalent to testing for association with Body Mass Index, BMI).
3. A test for association with *height controlling for weight*. (This analysis seems less natural, for reasons we discuss below).
4. A multivariate test of association with the bivariate phenotype (*height*, *weight*). Although this test can be performed in different ways, many approaches turn out to be equivalent. For example, one can test the global null of no association with either *height* or *weight* by either i) MANOVA, treating (*height*, *weight*) as a bivariate normal response and 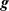 as an explanatory variable; or ii) ordinary least squares regression, treating 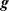 as a univariate response and (*height*, *weight*) as explanatory variables. For reasons discussed in [15], both these approaches lead to the same 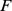 statistic (a result that also holds for more than two phenotypes), as can be easily verified empirically. [In R try, for example, g  =  rbinom(100,2,0.2); y  =  matrix(rnorm(1000), nrow = 100); summary(lm(g
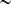
y)); summary(manova(y
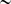
g)), and note the 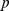 values and 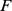 statistics are the same.]

**Figure 1:**
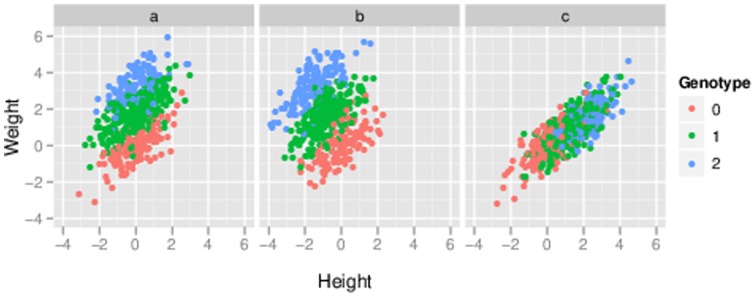
Illustration of three simple scenarios of association between genotype and a bivariate phenotype. All three scenarios involve positively-correlated bivariate response, which for concreteness we refer to as *height* (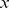-axis) and *weight* (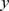-axis). Each point represents an individual, colored according to their genotype (0, 1 or 2 copies of the minor allele). A) A variant associated with *weight* but not *height*. Even though *height* is unassociated, it nonetheless clearly helps to consider *weight* and *height* jointly in testing for association: the separation between genotype classes in the two-dimensional space is substantially greater than the separation along the 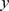 axis alone. In fact, here the most powerful analysis would be the test for association with *weight*, controlling for *height*. B) The minor allele decreases *height* but increases *weight*: it is an allele for being “short and fat”. Here the three genotype classes are much better separated in the two-dimensional space, than for either phenotype individually. Should one be lucky enough to encounter such a genetic variant, a multivariate test would be considerably more powerful to detect it than either univariate test. C) Here the minor allele increases *height*, and *as a result* increases *weight*, resulting in what we will call an “indirect” association with *weight*. In this case the separation of the groups in the bivariate space is no greater than the separation along the 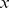 axis alone, and the most powerful analysis would be a univariate test for association with *height*. In all panels, the differences among genotype classes were deliberately made very large for clarity of presentation.

It is natural, and instructive, to consider under what circumstances each of these tests will be more powerful than others. Figure 1 illustrates three different scenarios, and discusses the most powerful test for each scenario. Even this simple bivariate setting produces some perhaps unexpected results. For example, naively one might have expected that if only *weight* is associated with genotype then the preferred test would be the univariate test of *weight*. However, as is clear from Figure 1a, the separation of the three genotype groups under this scenario is much better in the two-dimensional phenotype space than in the *weight* dimension alone, and so a joint analysis of the phenotypes should be more powerful. (Indeed, as we shall see later, in this case the test for *weight* controlling for *height* would be most powerful.) Conversely, one might naively expect that if both *height* and *weight* are associated with genotype then the multivariate test would be preferred. In some cases this is true (e.g. Figure 1b). However, in other cases the univariate test will actually be more powerful (e.g. Figure 1c). While these facts are arguably obvious in hindsight, in the author's experience they are easy to overlook in practice: indeed, most people seem to naturally assume that the main reason to do joint (multivariate) analyses is that the phenotypes may share a common set of underlying genetic associations, when in fact *multivariate association analyses are often most advantageous when not all phenotypes are associated with the genetic variant being tested!*

Even when we understand which tests will be most powerful in which scenarios, we then face a more fundamental problem: in practice, we do not know which association scenario, if any, holds for the variant we are considering, and so it remains unclear which test(s) to perform. A natural reaction to this is to perform several tests. However, while this is a reasonable strategy, it can be surprisingly tricky to interpret the results. For example, if a multivariate test gives a significant result, one does not know whether it is due to an association with *height* or *weight* or both. And although one could examine the univariate tests to assess this, this strategy is less than ideal for many reasons [16], particularly that it ignores the multivariate information that may have been crucial to detecting the association in the first place. There are also more subtle difficulties with interpreting the results of tests that control for certain variables. For example, while a test for association with *weight*, controlling for *height*, may appear to test for association with *weight*, in fact genetic variants that are associated with *height*, but not with *weight*, would also give significant results – or, more precisely, an excess of small 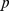 values compared with a uniform distribution – under this test! (To gain intuition into why, it may help to think of this test as akin to a test for association with BMI: any genetic variant associated with *height* but not *weight* would be associated with BMI.) Of course, all these issues will be magnified if we consider more than two phenotypes.

To summarize, the two main challenges confronting an analyst in this context are i) different tests have different power under different association scenarios, but we do not know which scenario we face in advance; and ii) the results of tests involving multiple variables may be difficult to interpret. In this paper we propose a framework that helps overcome both of these challenges. In a nutshell, the idea is to replace *testing* with *model comparison*. We define a collection of models, each of which corresponds to a different association scenario (such as those illustrated in Figure 1) and consider computing the support for each model relative to the “null” scenario of no association. We show how the support for each model is closely connected to the significance of a particular corresponding association test (e.g. tests 1–4 above), and so computing the support for each model effectively corresponds to performing a series of tests. However, viewing the outcome of each test as indicating the strength of support for a particular model greatly facilitates combining and interpreting results across tests. Although our framework uses Bayesian measures of evidence, we explore the close connection between these Bayesian measures and the outcome of standard likelihood ratio tests, and in particular, for normally-distributed phenotypes, we show that standard likelihood ratio tests effectively arise from the use of particular prior distributions.

The tools we use to implement the model comparison framework are not new, involving ideas and inference procedures from literature on Bayesian regression [17], [18] and graphical models [19]. However, the way we motivate these procedures is different than usual, and in particular we emphasize (apparently novel) connections between these inference procedures and traditional test-based analyses such as those outlined in 1–4 above. Indeed, we outline how the framework effectively includes all of the analysis approaches 1–4 as special cases, and provides a natural way to combine results from these different analyses. Our hope is that these connections will make the approach easier to digest for those more familiar with tests than with Bayesian graphical models.

### A unified framework

Consider assessing association between a single predictor variable 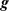 (e.g. a SNP genotype) and 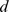 related variables 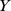, each measured on 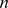 individuals randomly sampled from a population (so 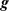 is an 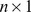 vector, and 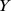 is an 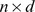 matrix). The size of 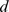 could affect choice of analysis methods, for both statistical and computational reasons; here we have in mind situations where 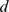 is reasonably small – in the range 2–10 say – although formally many of our results apply for all 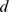. By “related” variables we mean variables that either are significantly statistically correlated with one another, or are approximately uncorrelated but plausibly mechanistically linked, and so could be expected to share some genetic influences. We return to the issue of which types of variables might benefit from being analyzed jointly in the Discussion. Although we are primarily motivated by genetic association studies, the framework described here also applies to multivariate association analysis more generally, at least to settings where 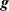 can be considered a randomized intervention. (Although genetic markers are not themselves randomized interventions in a conventional sense, it is often reasonable to treat them in this way due to Mendelian randomization; see e.g. [20].).

Simply stated, the aim of a multivariate association analysis is to identify which variables are associated with 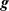 and which are not (keeping in mind that the answer may well be that “none of them are associated”). It turns out to be fruitful to consider subdividing the associated variables into two groups, “directly associated”, and “indirectly associated”. The distinction between these is made precise below in terms of conditional independencies, but, informally, an “indirect association” is an association that is mediated entirely through other measured variables. For example, in Figure 1c, *weight* is indirectly associated with 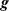 because the association is entirely due to the effect of 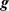 on *height*.

To formalize this, let 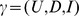 denote a partition of 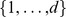 into disjoint subsets 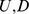 and 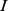, which represent, respectively, the variables that are unassociated, directly associated and indirectly associated with 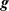. Let 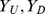 and 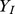 denote the corresponding columns of the matrix 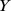 (so, for example, 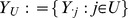). Since variables can only be indirectly associated with 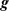 if some of them are also directly associated, we impose the restriction on 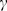 that if 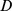 is empty then so must be 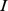. We associate with each partition 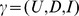 a probability model 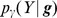 that satisfies the following conditional independence relations:

C_1_. 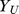 is independent of 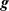.

C_2_. 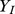 is conditionally independent of 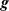 given 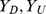.

(Although it is not required mathematically, in interpreting results we also implicitly assume that the variables in 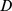 do not satisfy these conditions; that is, moving any subset of variables from 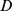 to either 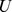 or 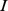 would negate one or both of 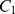 and 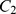. This is related to the concept of “faithfulness” in graphical models [21].) These conditions imply that 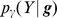 factorizes as: 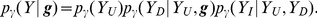
(1)


**Figure 2:**
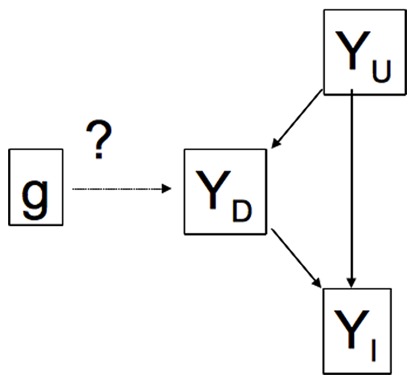
A graphical representation of the model corresponding to a partition 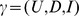
. Each of the nodes 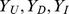 represents a subset of the measured phenotypes 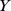. The simplest interpretation of the graph is as representing causal relationships among variables. In this interpretation a directed arrow from one node to another represents a direct causal effect, so, for example, the genotype has a direct causal effect on the variables 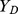, which in turn affects 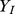. A more flexible interpretation is in terms of the conditional independencies among variables that would result from such causal network. The rules for obtaining these conditional independencies involve the notion of 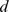-separation [63], which we do not go into here. Instead we simply note that the conditional independencies encoded by this graph include 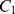: 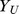 is independent of 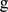; and 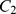: 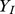 is conditionally independent of 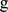 given 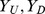 (because all paths from 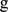 to 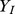 go through 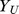 or 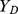). Note that the absence of any arrows in the direction from 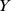 to 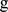 is justified by our treating 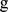 as a randomized intervention. (For those familiar with Directed Acyclic Graphical (DAG) models, here each 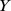 node represents a collection of variables, and we allow for arbitrary correlations among the variables within each node. Thus, in a full DAG representation arrows would exist between all pairs of 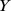 variables: arrows between variables in different nodes would go in the direction indicated by the figure. Arrows between variables within a node could go in any direction, subject to the constraint that the resulting graph must be acyclic.).

[A note on notation: throughout the paper all distributions are conditional on 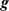, but some of these conditional distributions do not depend on 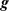, a fact that we indicate by dropping 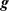 from the notation. Thus, for example, we use 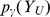 for 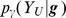 to indicate that this conditional distribution does not depend on 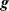.] Note that the usual global null hypothesis, which is that 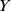 is independent of 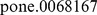, corresponds to the partition with all variables in 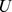, i.e. to the partition 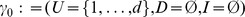. We consider specification of suitable distributions 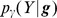 in more detail below; for now we consider them to be given, and fully specified (i.e. no unspecified free parameters). The relationships among 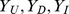 and 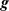 can be visualized graphically as in Figure 2.

We assume that some (unknown) value of 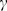 gave rise to the observed data, meaning that 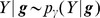, and treat 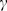 as a parameter to be inferred. Since 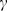 identifies which coordinates of 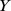 are associated with 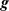, inferring 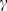 can be viewed as the main goal. We perform inference for 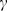 using Bayesian methods, which involves specifying a prior distribution 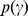, and computing the posterior distribution using 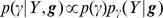. Choice of appropriate prior distribution will be context-dependent, and is discussed further below.

The posterior distribution for 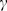 contains all the information needed for both testing for and interpreting associations between 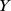 and 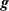. For testing, the overall evidence against the global null hypothesis 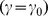 is given by the probability that this hypothesis does not hold, 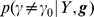. For interpretation, the posterior on 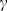 quantifies the strength of the evidence (posterior probability) that any particular combination of variables is directly or indirectly associated with 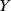. For example, the marginal posterior probabilities for each coordinate being in 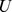, 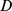, or 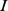 seem a particularly useful summary, and take the form 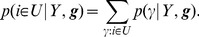
(2)


Because each value of 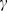 effectively defines a different statistical “model”, performing inference for aspects of 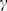 in this way, by summing over models, is often referred to as “Bayesian model averaging” (BMA).

While there are many possible arguments for a Bayesian approach to inference, here we find it particularly convenient that, through the use of BMA, it has the potential to answer questions about aspects of 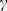 even when the actual “true” value of 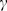 may be difficult to infer reliably. For example, suppose that the data strongly suggest that 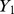 is directly associated with 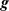; but are relatively uninformative about other coordinates of 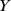. In this case the posterior distribution on 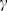 would be diffuse, spread out over a large number of partitions, but the posterior would nonetheless be informative because it would be restricted to partitions in which 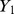 is in 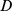 (so 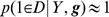). In addition, the Bayesian framework ensures that answers to inter-related questions are consistent with one another. For example, the posterior probability that any *particular* coordinate of 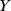 is associated with 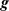 will always be less than the overall posterior probability that *at least* one coordinate of 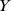 is associated. In other words, the evidence against the global null is always greater than the evidence against the univariate null for any given coordinate, which it logically should be because the global null hypothesis implies all univariate null hypotheses. In contrast, use of 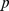 values from standard tests to measure evidence does not enjoy this property: performing standard univariate and multivariate tests can yield smaller 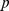 values against the univariate null than against the global (multivariate) null.

#### Specifying 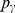


Implementing the above inference approach involves specifying a model 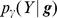, for each possible value of 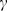. This is a large number of models even if 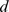 is only moderate. In this section we outline a simple strategy for specifying all these models, which involves explicitly specifying only two models, and then deriving all other models from these. This approach is analogous to [22], which considers deriving a large number of graphical models from specification of the single model corresponding to a complete graph.

The two models that must be specified are those corresponding to the “global null”, in which all variables are in 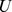, and what we will call the “full alternative”, in which all variables are in 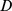. We let 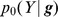 and 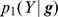 denote these two probability distributions. Suitable forms for 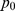 and 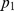 will be context-specific; in following sections we consider specific choices for 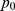 and 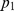 when 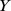 can be assumed multivariate normal within each genotype class.

Recall that 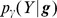 factorizes as: 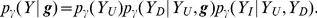
(3)


Now make the following two assumptions:

A_1_. The distributions that do not depend on 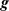 (the first and the last) are the same as under the null 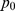;

A_2_. The distribution that does depend on 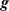 (the second) is the same as under the full alternative 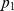.

Then 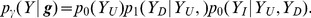
(4)


Thus assumptions *A1–A2* yield a model 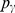 for each 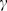, using only 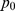 and 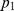.

Besides simplifying the problem of specifying the many probability distributions 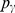, the assumptions (*A1–A2*) leading to (4) may be viewed as desirable in themselves, since they ensure that all the distributions 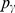 are in some sense “consistent” with one another, agreeing on some parts of 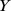 where we might wish for them to agree. For example, suppose we consider two different partitions, 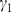 and 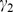, in both of which the variable *height* is unassociated with 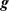. Then the assumptions *A1–A2* ensure that the marginal distribution of *height* will be the same under both 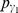 and 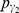 (and, as a result, observing only the distribution of heights in the samples would tell you nothing about whether other phenotypes are associated with 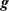).

#### Connections with testing

We now describe the connection between the support for each partition 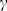 in the above framework and standard tests for association.

The support for partition 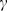, relative to the global null hypothesis 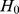, is given by the likelihood ratio, or Bayes Factor (BF),
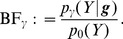
(5)


Large values of 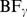 indicate support for partition 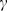 compared with the null. Indeed, in terms of traditional hypothesis testing, a test that rejects 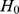 if 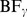 exceeds some threshold is the most powerful test of its size under the alternative hypothesis 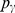 (by the Neyman-Pearson lemma; [23]).

Now, noting that the null distribution 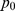 can be factorized as 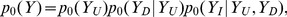
(6)and taking the ratio of (4) to (6), we obtain



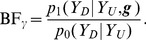
(7)Note the attractive intuitive interpretation of the right hand side of (7): it is itself a likelihood ratio, or BF, for comparing a model where 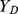 depends on 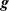 given 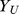 with a model where 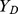 is independent of 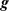 given 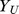. That is, 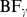 is effectively a test statistic for whether 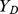 is associated with 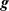, controlling for 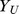.

Thus expression (7) establishes a link between the support for each partition 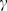 (vs 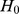), and commonly-used tests of association. In words:

[Support for γ  =  (*U*, *D*, *I*) vs *H*_0_]  =  [Support for *Y*_D_ being associated with ***g *** given *Y*_U_].

Put another way, the support for each partition 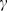 corresponds to a test in which some subset of the variables (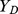) is treated as the response variables, another subset (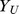) is controlled for, and the remaining subset (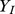) is ignored. Our derivation assumes that the 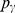 are fully specified, and so applies to Bayesian tests, which integrate over prior distributions on free parameters, but not directly to standard likelihood ratio tests, which maximize over free parameters. However, when 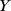 is modeled as multivariate normal, these two types of tests can be very closely related, as is made explicit in Proposition 1 below.

To give concrete examples, each of the tests 1–4 mentioned in the Introduction can now be seen to correspond to the support for a particular partition 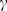:

- The univariate test of *height* corresponds to support for a direct association with *height* with an indirect association with *weight* (*height* 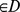 and *weight* 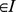).
- The test for *weight* controlling for *height* corresponds to support for a direct association with *weight* and no association with *height* (*weight* 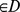, *height* 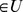).
- The test for *height* controlling for *weight* corresponds to support for a direct association with *height* and no association with *weight* (*height* 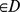, *weight* 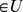). This partition, and hence this test, seems less natural because we might expect that any genetic variant affecting *height* would also affect *weight*.
- The general multivariate test corresponds to support for a direct association with both *height* and *weight* (*height* and *weight* 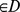).

Although deriving the relationship (7) is algebraically trivial, the relationship itself is conceptually non-trivial. In particular, for different 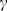, the tests that occur on the right of the equation are conceptually very different from one another, involving different null hypotheses. For example, the null hypotheses for the univariate test of *weight* (“ *weight* is unassociated with 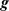
”) and for the univariate test of *height* (“*height* is unassociated with 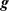
”) are different, and tests of these hypotheses depend on different parts of the data, making them appear difficult to compare. Equation (7) shows how these various tests can be viewed within a single framework by thinking of each of them as a test for a particular multivariate alternative hypothesis against the global null hypothesis.

The link between partitions and tests also provides a helpful indication of which tests will be (asymptotically) most powerful under which circumstances. For example, if only one of the phenotypes (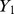 say) is associated with 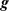, and all others are unassociated, then the most powerful test will not, in general, be the univariate test for association with 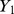, but will instead be the test for association with 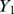 *controlling for the other phenotypes*. Conversely, even if *all* the phenotypes are associated with , if only 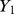 is *directly* associated then the univariate test of 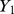 will be the most powerful. While these observations may be regarded as trivial in hindsight, they nonetheless emphasize something that is otherwise easy to forget: that simultaneous analysis of multiple related phenotypes may be helpful even if – indeed, particularly if – only one of the phenotypes is associated with a particular genetic variant.

#### Testing the Global Null

In a typical genetic association analysis the vast majority of genetic variants will not be associated with any of the measured phenotypes, and so it is natural to focus, initially, on whether (for each genetic variant 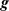) the data suffice to reject the global null hypothesis 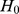.

The overall evidence against the global null 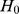 is summarized by the overall Bayes Factor, which we denote as 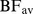 (av representing average), 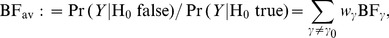
(8)where the weights 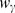 are proportional to the prior distribution 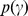 and normalized to sum to 1 [i.e. 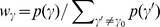]. In a Bayesian analysis, the posterior probability of 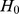 would be computed from the prior probability on 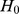 (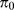 say) and 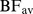 using



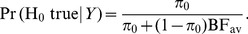
(9)If a frequentist test of 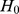 is desired, then 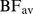 could be used as a test statistic, and 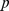 values estimated by simulation/permutation.

Note the attractive intuitive interpretation of (8): 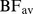 is a weighted average of the Bayes Factors from the many different possible tests one might consider. Thus, if one prefers, one can think about specifying weights for different tests, rather than specifying a prior on 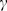. For example, performing only the full multivariate test, which corresponds to the partition with all variables in 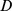, corresponds to putting weight 1 on that partition, and no weight on any other partitions. We use 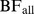 to denote this Bayes Factor: 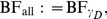
(10)where 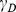 is the partition with all variables in 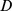. Also, performing only the univariate tests corresponds, intuitively, to putting equal weight (1/d) on the 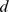 partitions that correspond to each of the univariate tests:
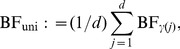
(11)where 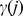 denotes the partition corresponding to the univariate test of variable 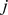 (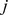 in 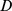 and all other variables in 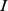).

When viewed in this way the standard multivariate test and univariate tests correspond to rather strong assumptions, since they assign 0 weight to many partitions, and thus rule them out *a priori*. In general it would seem preferable to avoid such restrictive assumptions, and place at least some weight on all (or most) partitions. On the other hand, equal weight on all non-null partitions also has some unattractive properties: for example, for moderate 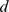, this would put almost no weight on models in which a single variable is associated with 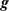. One alternative for a “default” prior (where we have in mind a prior to be implemented in software for general distribution) would be to place a uniform prior on the number of variables associated with 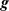 (conditional on at least one variable being associated). Specifically, if 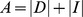, then conditional on 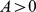 we assume that 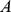 is uniform on 1 to 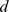; further, conditional on 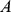 we assume that 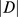 is uniform on 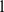 to 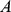. Finally, if the coordinates of 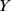 are assumed to be exchangeable, then given 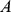 and 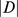 all partitions 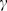 with that 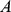 and 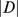 are equally likely, which yields 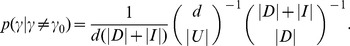
(12)


Under this prior, the expected value of 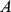 is 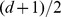, and so by symmetry the prior probability that any particular variable is associated is 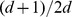, which equals approximately 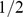 for moderate 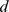. For larger values of 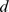 a prior that more heavily favors smaller values of 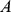 might be more appropriate. If the coordinates of 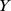 are not exchangeable then this prior could be improved upon. For example, if 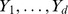 reflect temporally or spatially ordered observations then it will typically be desirable to put more weight on partitions in which consecutive variables fell into the same category (e.g. 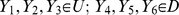 would get more prior weight than 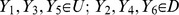). In other cases there may be physical relationships among the 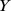 variables that affect the prior on partitions. For example, if we are interested in *height* and *weight*, it seems quite plausible that a genetic variant that affects *height* would have a knock-on (indirect) effect on *weight*, but substantially less plausible that a genetic variant affecting *weight* would have a corresponding knock-on effect on *height*. However, quantifying this kind of information may be difficult and tedious, especially if 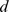 is large, and so even though such issues are relevant in principle, it is undoubtedly easier, and perhaps generally not too harmful, to ignore them in practice.

While any particular prior choice of weighting scheme is likely to appear somewhat arbitrary, we view (12) as no more arbitrary than – and, indeed, generally preferable to – limiting analyses to either a single multivariate test or to the 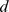 univariate tests. This said, where possible it would be preferable to take a more hierarchical or “data driven” approach. For example, in genome-wide association studies, provided sufficiently many associated SNPs can be identified, we can “learn” about which phenotypes tend to share genetic factors, and hence effectively learn an appropriate prior for 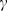 (i.e. “Empirical Bayes”). We illustrate this in our data analysis below.

### Multivariate normal phenotypes

In this section we describe a way to implement this framework for the important special case where 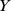 is multivariate normal within each genotype class. We also formalize the mathematical connection, in this special case, between Bayes Factors for each partition, and standard likelihood ratio tests. This material is necessarily more algebraic, and of most interest to those applying these methods in practice, and to those interested in the formal mathematical connections. Since this section does not introduce any important new concepts, it could be skipped on a first reading by those keen to see examples and results.

For multivariate normal outcomes we use Bayesian Multivariate Regression (BMVR) [24]–[26] to specify the null distribution 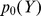 and general alternative distribution 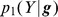. Our treatment here owes much to helpful material in [18]; [27] also provides particularly relevant background.

The standard multivariate regression model is 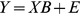
(13)where 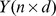 is a matrix of 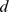 outcome measurements (response variables) on each of 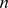 individuals; 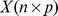 is a matrix of 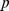 covariates (explanatory variables) measured on the same individuals; 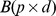 is a matrix of unknown regression coefficients relating the outcomes to the covariates; and 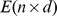 is a matrix of error terms, whose rows we assume to be independent and identically distributed as 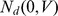 for some unknown covariance matrix 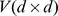.

Bayesian multivariate regression requires specification of prior distributions for the unknowns 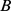 and 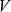. We use the conjugate prior for 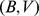, which is not only computationally convenient, but, as we will see later, leads to Bayesian procedures that have some attractive properties and close connections with traditional testing procedures such as MANOVA. Specifically, the conjugate prior for 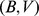 is 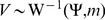
(14)

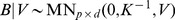
(15)where 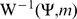 denotes the inverse Wishart distribution with (inverse) scale matrix 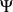 and degrees of freedom 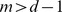; and 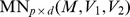 denotes the matrix normal distribution on 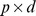 matrices, with mean 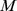, and covariance matrices 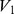 (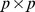) and 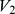 (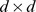).

For readers unfamiliar with the matrix normal distribution, note that if 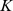 is a diagonal matrix, as we assume here, then the matrix normal prior (15) for 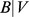 reduces to independent multivariate normal priors on the rows of 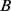, each having covariance matrix a scaled version of 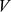, the covariance of the 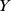
s. Specifically, if 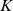 is the diagonal matrix with diagonal elements 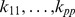 then the prior on the 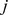
th row of 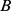 is 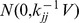.

Use of this prior has been criticized on the grounds that it imposes overly-restrictive constraints on the prior covariance of 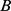 (see [26], p253, who cites [28]). However, in the absence of specific prior information to the contrary, this relationship may be appropriate. For example, consider a situation where two outcome variables 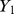 and 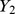 are positively correlated with one another. Then, the above prior implies that any genetic variant that increases 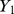 is more likely to increase (rather than decrease) 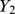; and that conversely any variant that decreases 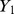 is more likely to decrease (rather than increase) 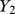. Note that all possible combinations of increase/decrease are possible, but some are considered *a priori* more likely than others.

Using these priors the marginal likelihood for 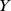, 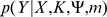, can be computed analytically (see, for example, [18], equation (52)). Specifically, 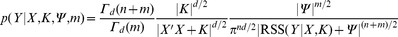
(16)where 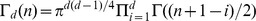 is the multivariate Gamma function, and
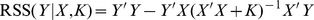
(17)is a Bayesian analogue of the residual sums of squares matrix.

The distribution (16) for 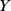 is a matrix-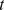 distribution [29]–[31]. Here we will denote this distribution by 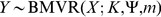
(18)to emphasize that it arises from performing a Bayesian MultiVariate Regression of 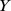 on 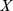.

#### Specification of 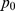 and 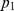


We now specify the two key distributions, 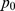 and 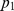, from which all 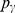 will be derived (via equation (4)). We take the global null model 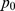 to be BMVR on an intercept alone, and the full alternative model 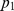 to be BMVR on an intercept and 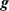: 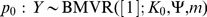
(19)

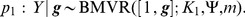
(20)


Here 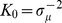 and 
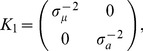
(21)where 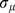 and 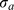 are hyperparameters that control the variance of the prior distributions on, respectively, the intercept parameters and the effect size parameters associated with 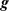.

With these choices of 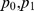, the Bayes Factor for partition 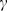, given by (7), has a particularly intuitive form. Indeed, due to special properties of the priors assumed for the BMVR, both the numerator and the denominator of this expression are also BMVRs. Specifically, from Proposition S.4.1, in Section S.4.2 of Supplementary Information S1, 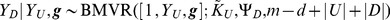
(22)

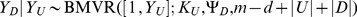
(23)where we have assumed for simplicity that 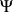 is diagonal, and where 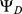 denotes the submatrix of 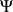 corresponding to coordinates in 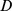, 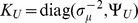, and 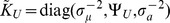. Since 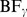 is the Bayes Factor for comparing model 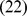 with 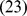, it is, in a precise sense, the BF for comparing a model in which 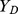 is a regression on both 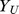 and 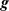 against a model in which 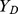 is a regression on 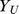 alone. Further a simple analytic expression for 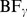 is easily obtained by taking the ratio of 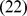 and 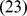, each of which has an analytic expression of the form (16).

#### A limiting prior for the hyperparameters, and connections with likelihood ratio tests

The Bayes Factor 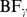 depends on hyperparameters 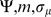, and 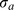. Here, and for the remainder of the paper, we consider the limits 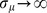, 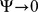 (although in some settings other priors may be preferable; see Practical Issues, below, for discussion). The resulting Bayes Factors, which we denote 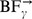, have very close connections with standard frequentist tests based on ordinary multivariate regression models, as we now discuss.

In the limits 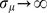, 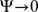 the Bayes Factor 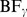 tends to 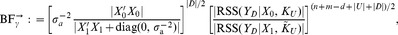
(24)where 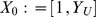, 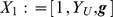, and diag(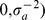 is the 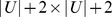 matrix with 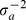 at position 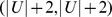 and zeros elsewhere.

To state the relationship between 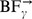 and traditional tests, let 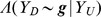 denote the standard likelihood ratio statistic from a normal regression-based test of whether 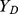 is associated with 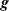, controlling for 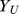. That is, 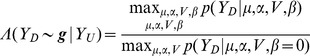
(25)where 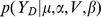 is given by the normal multivariate regression model
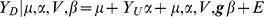
(26)with error terms 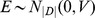.

The following proposition and notes explore important properties of 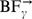, including its relationship with the likelihood ratio statistic, and its invariance to measurement scale of the phenotype.

**Proposition 1** *The Bayes Factor* 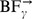 *is related to the likelihood ratio statistic* 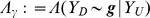 *by*

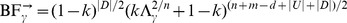
(27)
*with* 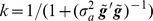, *where* 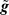 *denotes the vector of residuals from OLS regression of* 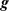 on 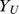 (including an intercept).

A proof is provided in Supplementary Information S1 (Section S.2).

**Corollary 1** 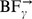 *enjoys the following properties: [a)]*

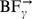
 *is invariant to invertible affine transformations of* 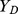 *and/or* 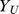.* That is, if* 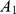 *and* 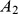 *are any invertible* 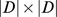 *matrices, and* 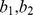 *are any* 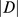 *-vectors (* 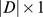 *matrices) then* 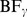 *computed using the transformed phenotypes* 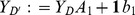 *and* 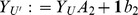 *is the same as using the original phenotypes* 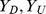 *(This follows from Proposition 1 because *.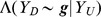 *also enjoys this property.).*
- *For any fixed* 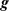 *and* 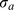 *,* 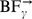 *is monotonically increasing with* 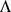 *(as* 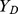 *varies)*.
- *For fixed* 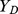 *, if for each SNP* 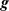 *we use* 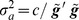 *, for some fixed* 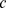 *, then* 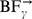 *will rank the SNPs in the same way as* 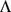.

#### Note 1

*Property a) above implies that* 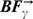 *is invariant to choice of coordinate systems for* 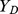 *and* 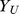 *, and in particular to changing units of measurement (e.g. measuring height in meters vs inches). As a special case, consider the Bayes Factor* 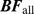 *(10) for testing whether all the variables are directly associated with* 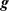 *. Property a) implies that* 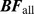 *is invariant to choice of coordinate system for* 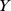 *. Thus, in the settings illustrated in* *Figure 1* *, the result of an association test would be unchanged by rotating the figures.*

*Property b).* suggests a certain amount of robustness to choice of 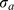 *. In addition, if we accept* 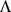 *as a reasonable measure of the association information in the data, then b) also provides some level of general reassurance that the priors being used to compute* 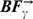 *do not overwhelm this information. Some might say that the priors are “uninformative”, or that they “allow the data to speak”*

*Property c) implies that, if the condition on* 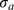 *holds, then ranking SNPs by the* 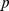 *value from* 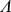 *would produce the same rankings as a Bayesian analysis that assumes the stated limiting priors. Thus, this property gives the prior assumptions that implicitly underlie traditional analyses, generalizing the univariate result linking* 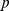 *values and Bayes Factors in* [*32*]. *In the special case where* 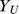 *is empty,* 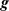 *is simply the mean-centered genotypes, and so the condition on* 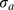 *becomes* 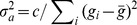 *. This condition, which is the same as the condition in* [*32*], *corresponds to assuming that effect sizes of non-null SNPs tend to be larger for rare SNPs (those with a low frequency of one allele). Of course, within the Bayesian framework it is easy to make a different assumption (e.g. that* 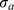 *is the same across SNPs) if one prefers. A further connection between our Bayes Factor and the approximate Bayes Factor from* [*32*] *is given in Note S.1.1 in Section S.1 of Supplementary Information S1.*

#### Note 2

*It is an elementary, although perhaps surprising, result (see* [*15*] *for example) that* 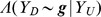 *is equal to* 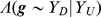 *: that is, in a normal regression setting, when testing for association between* 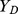 *and* 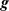 *using a likelihood ratio statistic, it does not matter which way around one does the regression. Thus the above results also link the Bayes Factor with the test statistics from the “reverse” regressions,* 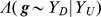.

### Practical Issues

#### Prior on (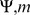)

Proposition 1 above considers properties of the Bayes Factors that arise in the limit 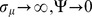. In our applications below, which all involve relatively low-dimensional phenotypes (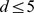) we make use of this limiting Bayes Factor, together with the limit 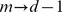, which is the limit of a proper prior (the inverse Wishart prior on 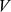 is proper for 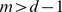). For larger 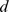 we expect it will be preferable to use different priors, particularly for 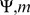 which determine the prior on the error variance-covariance matrix 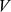. In low dimensions the data will be highly informative about 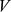, and we expect inferences to be relatively robust to choice of 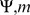. However, for higher dimensions it is usually desirable to regularize estimates of covariance matrices, and so a prior that effectively regularizes 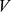 seems likely to be preferable. (At the simplest level, using 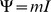 with 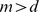 will provide some regularization; more complex prior structures that provide more sophisticated regularization may be more preferable still.) We view the application of this framework to higher dimensional data as a potential area for future research.

#### Prior on 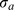


Computing the Bayes Factors 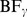 also requires specification of 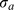, which controls the expected size of the effect of 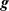 on the elements of 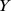 under 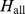. This need to specify an effect size parameter is shared with the corresponding univariate analysis. In practice we usually average results over multiple values of 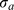, which corresponds to assuming a discrete prior on 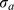. By using a (possibly weighted) combination of smaller and larger values of 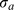, we can allow the prior on effect sizes to be concentrated on small values (small 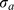) whilst not ruling out the possibility of large effects (large 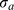). In the univariate context this averaging strategy provides a very flexible set of prior distributions. However, in the multivariate context this prior is more restrictive than one might like, because it assumes that the value of 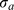 is shared across all phenotypes. This effectively ties together the prior on the effect sizes on the different phenotypes, and limits the prior weight on a genetic variant having a large effect on some phenotypes and small effects on others. Again, developing methods that can deal with more flexible prior assumptions is a potential area for future research.

In practice, the need to specify suitable values for 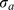 is perhaps the aspect of prior specification that most users will find hardest. For practical guidance (in the univariate case, but which also applies to multivariate analyses) see [33]. In our real-data application below, we used the strongest observed associations to help guide selection of suitable “data-driven” values of 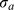, and this may also be a helpful general strategy.

#### Computation

In Supplementary Information S1 (Section S.1) we give an algorithm, and R code implementing efficient calculations of 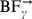 for all partitions and all SNPs in a genome-wide association study.

For modest values of 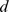 (and large 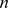) the overall computational burden of the multivariate analysis is not appreciably greater than performing 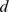 univariate tests. One reason for this is that, as shown in the Supplementary Information S1 (Section S.1, Lemma S.1.1), 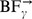 depends on 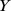 and 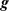 only through the following summary statistics: 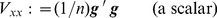
(28)

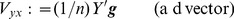
(29)

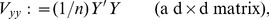
(30)which need be computed only once for all partitions 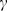. Computing these summary statistics in a genome-wide association study involving 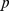 SNPs on 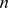 individuals requires computation 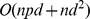. Then, for each partition 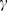, computing 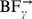 for all SNPs takes less than 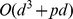 (there are matrix decompositions that are 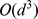 that need to be performed only once, and then the computations for each SNP are linear in 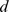). Thus the total computation for 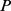 partitions is 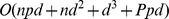, and if 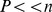 then this is dominated by the 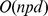 term that also applies to 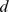 univariate analyses.

Of course, the number of partitions 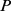 grows quickly with 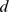 (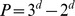), and for 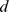 greater than about 15 computing 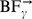 for all partitions will be impractical. In this case computational approximation methods may help: for example, Markov chain Monte Carlo could be used to sample from the posterior distribution of 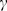. However, for 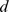 of this size there may also be statistical issues that need addressing to make these methods suitable for routine application (e.g. choices of priors for 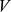 and 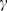 may need revisiting, as discussed above).

Even for smaller values of 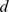 it may be tedious to compute all partitions for a large number of SNPs in a genome-wide association study. One strategy for reducing the computational demands of a GWAS is to perform a two-step analysis, the first step being a computationally quick heuristic to identify a list of promising SNPs, and the second step being a more comprehensive analysis of these promising SNPs. For example, in our application below, we perform the first step using a simple multivariate test and all 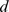 univariate tests. This corresponds to considering just 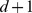 possible partitions, which is feasible even for large 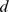. Then we analyze promising SNPs in detail by considering all possible partitions.

#### Missing data, and incomplete access to data

As noted above, 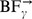 depends on 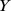 and 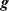 only through the summary statistics (28)–(30). In many cases these summary statistics, or approximations to them, can be readily computed even with incomplete data – for example, if some phenotype data are missing, or if the full phenotype and genotype data from the original study are hard to obtain, which is often an issue in GWAS. For example, the first two quantities can be approximated from an estimate of the SNP minor allele frequency, 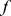, and a 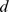 vector of the usual effect size estimates 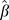: 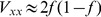
(31)

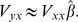
(32)


The third summary statistic, 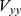, estimates the covariance matrix of the phenotypes, which can also be approximated in various ways. In an application below (“Global Lipids GWAS”) we show how an approximate analysis can also be performed using only 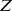 scores, an allele frequency estimate, and a sample size (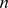) for each SNP.

If some phenotype data are missing, then the elements of both 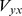 and 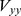 can be approximated using only those individuals for which the relevant phenotypes are available. For example, the elements of the vector 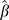 can be computed separately for each phenotype, using only the non-missing values; and similarly for the elements of 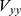 using pairs of phenotypes (e.g. using the cov function in R with use  =  “pairwise.complete.obs”). We expect such approximations to be adequate, at least in settings where the amount of missing data is modest, and the data are missing at random. (Note that 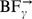 depends on sample size 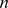, which varies among phenotypes if some phenotype data are missing; in this case we suggest using the smallest value of 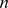 across phenotypes as the value for 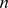 in the BF calculation.)

## Results

### Simulations

#### Simple bivariate simulations

To illustrate some key points, we begin with simple bivariate simulations in which phenotypes 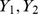 are associated, in varying ways, with SNP genotypes 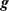. Each simulation scenario is defined by three parameters, 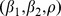, which denote, respectively, the genetic effects on 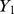 and 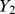, and the correlation coefficient of the residuals. Specifically, we simulated datasets of 1,000 individuals, where for each individual 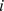 we simulated a genotype  Binomial(2,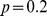), and bivariate phenotypes 
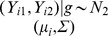
, where 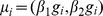 and 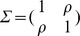.

We fixed 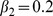, and considered two different levels of correlation (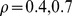) and 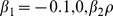. These values of 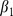 correspond, respectively, to 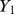 being Directly associated, Unassociated, and Indirectly associated with 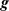.

For each dataset we compared the Bayes Factors 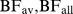 and 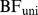 (defined at (8), (10) and (11)) with one another, and with a “reference” Bayes Factor which is the BF for the “true” partition (which varies according to the simulation scenario). For example, when 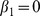 the reference BF is the BF for the partition in which 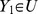 and 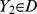. All BFs were computed with 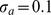 (results are qualitatively robust to this choice of 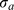).

**Figure 3:**
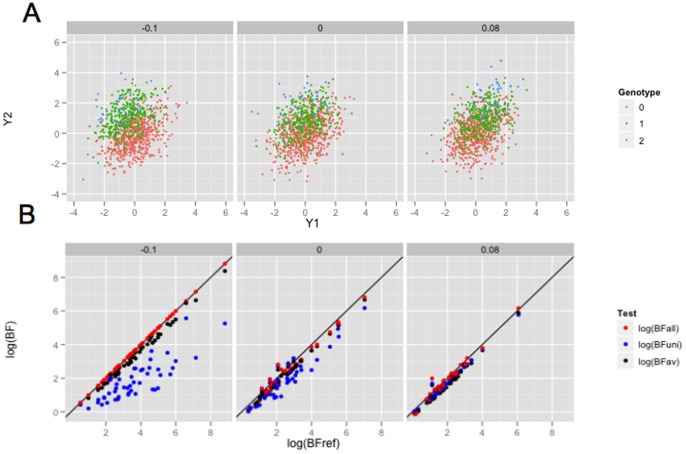
Comparison of Bayes Factors in simple bivariate simulations, correlation  = 0.4. The upper panel shows a typical simulated dataset under each of three scenarios (see text), but with effect sizes increased 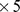 to aid clarity; each dot represents a single individual, colored according to genotype. Note that in the middle scenario only 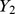 is associated with genotype. The lower panel compares 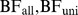 and 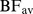 with a reference BF, which is the theoretical optimal for that simulation scenario. Thus one can see not only how the BFs compare with each other, but also the extent to which they lose compared with the optimal. Each point represents the results from a single simulation, and each simulation is represented by three points.

**Figure 4:**
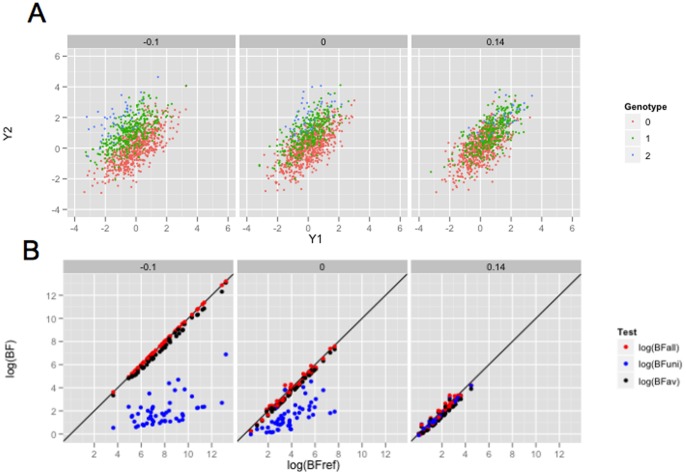
Comparison of Bayes Factors in simple bivariate simulations, correlation  = 0.7. See caption to Figure? 3 for more details.

Figures 3 and 4 show illustrative simulated datasets under each scenario (panel a) and results of the BF comparisons (panel b). We emphasize three features of the results. First, the “reference” BF is generally as large, or larger, than the other BFs computed. This is reassuring, as it indicates that the support for the “correct” model/partition is generally as large or larger than support for other models. Second, the BFs that correspond to multivariate tests (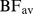 and 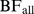) are sometimes appreciably (orders of magnitude) larger than the BF that corresponds to univariate tests (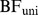), while the converse is not true. Thus, in the settings considered here, **the potential gain from performing multivariate tests is much higher than any potential loss.** Note also that, as expected from previous discussions, the multivariate BFs provide a stronger association signal than the univariate BFs even when only one phenotype is associated with 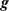 (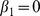, middle column). Finally, 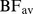 and 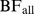 generally do not vary greatly from one another in these two dimensional examples; we would expect the difference to be greater in higher dimensions, as discussed below.

#### Five-dimensional power simulations

Next we performed some conventional “power” simulations in scenarios involving 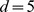 phenotypes. We simulated data under six different scenarios. In the first two scenarios only one phenotype was associated with 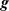; in the next scenario all the phenotypes were associated with 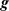, but only one was directly associated; in the remainder multiple phenotypes were directly associated with 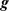. The six scenarios were as follows:

- Independence: phenotypes were independent, one phenotype associated with 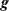.
- One variable directly associated, rest unassociated: 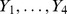 are independent of one another and of 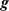; 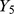 is correlated with all four other phenotypes, and with 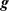.
- One variable directly associated, rest indirectly associated: 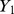 is associated with 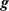; 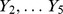 are simulated by adding various amounts of (independent) noise to 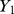, and so are also indirectly associated with 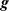.
- Multivariate 1: a more complex scenario in which 2 variables are directly associated with 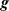, two are unassociated, and one is indirectly associated. (Variables are correlated at varying levels.)
- Multivariate 2: two variables are directly associated and the rest are unassociated. (Variables are correlated at varying levels.)
- Latent factor: a single latent factor is simulated that is associated with 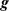. Then 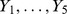 are simulated by adding various amounts of noise to 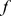. Under this scenario, all variables are directly associated with 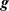.

The code used to simulated each scenario is given in Supplementary Information S1 (Section S.3).

**Figure 5:**
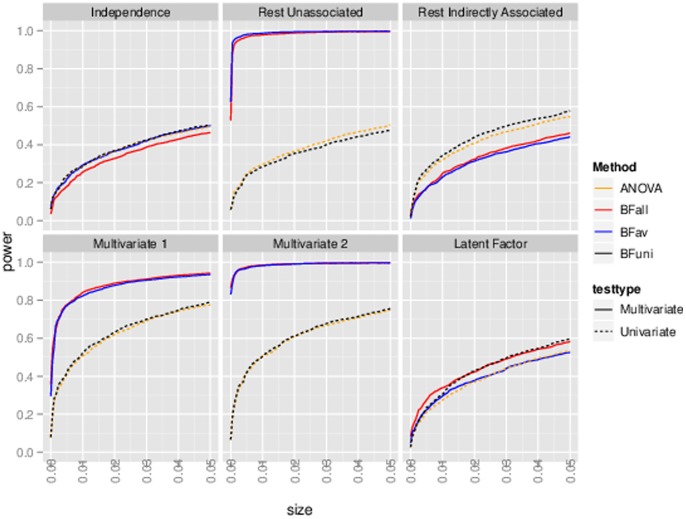
Power comparison of different test statistics under different simulated (five dimensional) multivariate scenarios. Each line shows the power vs size for a different test statistic; the univariate tests (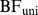 and ANOVA) are indicated by dotted lines. See main text for details of each simulation scenario and the test statistics compared.

For each scenario we simulated 10,000 “alternative” data sets with a genotypic association and 10,000 null datasets with no genotypic association (but with the covariance of the error terms the same as under the alternative). Each dataset had 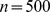 individuals. For each test statistic, at any given rejection threshold, we estimated the size (type I error rate) from the test statistic values on the simulated null data, and the power from the test statistic values on the simulated alternative data. Figure 5 plots power against size for each test statistic.

In addition to the three BFs considered in the previous section (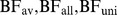) we also used as a test statistic the minimum 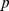 value from the 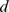 univariate tests of association (from regressing 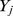 on 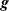); marked as ANOVA on the plot. This test statistic is highly correlated with 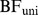, as should be expected since both are based on the 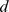 univariate association analyses. Note that, by Proposition 1, 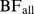 produces identical results here to a standard multivariate likelihood ratio test (and indeed to MANOVA, as implemented in R).

As expected, different tests have better power in different scenarios. We emphasize two general features of the results. First, the relative performance of the tests is, reassuringly, as expected from previous discussion. For example, the univariate tests are more powerful when the true model is that one phenotype is directly associated and the rest are indirectly associated, whereas the multivariate tests are more powerful when one phenotype is directly associated and the rest are unassociated. Second, in as much as one can draw general conclusions from these results, it seems that the multivariate tests tend to be more powerful than the univariate tests. The clearest exception to this is the rather special case where one variable is directly associated and the rest indirectly associated, which was specifically chosen for inclusion here because it is the case where the univariate test is optimal. In addition, consistent with the bivariate simulations, the potential increase in power of the multivariate test over univariate tests tends to be greater than the potential gains of univariate tests over multivariate tests.

### Global Lipids GWAS data

To investigate the potential for multivariate association analysis methods in practice, we applied the framework outlined here to GWAS data from the Global Lipids consortium [34]. These data comprise more than 100,000 individuals of European ancestry (obtained from 46 separate studies), genotyped genome-wide on large-scale SNP genotyping chips, and phenotyped for four blood lipids phenotypes: total cholesterol (TC), low-density lipoprotein cholesterol (LDL-C), high-density lipoprotein cholesterol (HDL-C) and triglycerides (TG). The original univariate association analyses in [34] reported a total of 95 SNPs as being associated with one or more of these phenotypes.

For these data we have access only to summary statistics, and not the raw genotype and phenotype data. Specifically, for each of the four phenotypes we have access to univariate 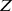 scores from the meta-analysis of [34]. Consequently, to apply our framework we had to make approximations. These are described, together with other details of the analysis, in Detailed Methods below. Since in GWAS access to summary data is often substantially easier to arrange than access to raw data, these methods for applying the framework in this setting are practically very important.

Before conducting a genome-wide analysis, we first applied our framework to the 95 associated SNPs identified in [34]. Rather than specify subjective prior distributions for the partitions 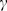 and effect size variance 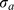, we instead took an empirical Bayes approach, estimating the relative frequency of different partitions and effect sizes from the data. Specifically, we estimated weights 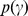 and 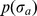 (for a grid of values of 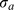) by maximum likelihood estimation. Because the 95 SNPs were selected to be the most strongly associated SNPs, the estimates of 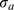 will likely be biased upwards. However, this data-driven approach seemed preferable to fixed subjective specification of 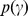 and 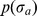. Given the estimated weights, 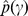 and 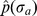, we computed the posterior distribution on the partition 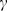 for each SNP. This allows us to assess, for example, which SNPs are associated with which phenotypes. See Detailed Methods for further details of the methods used.

Our results suggest that most of the 95 SNPs are actually associated with all four phenotypes. Indeed, 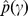 assigned a total probability of 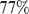 to models in which all four phenotypes were associated (either directly or indirectly) with the SNP. Almost all the remaining probability (21%) was assigned to models in which three of the outcomes were associated (8%, 8% and 5% assigned to models in which LDL, HDL and TG were unassociated respectively, with effectively no weight assigned to models in which TC was unassociated). Of the 95 SNPs, only 11 had 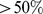 probability of being unassociated with at least one trait (Table 1).

**Table 1:** Table of genes from Global Lipids study [34], that, in our analysis, are best classified as being unassociated with one of the four lipid traits. (All other genes were best classified as being associated with all four lipid traits).

Gene	Marginal (univariate)	Unassociated Lipid Trait	Posterior
	associated traits	(multivariate)	Probability
LPL	TG:HDL	LDL	0.99
MLXIPL	TG:HDL	LDL	0.98
LIPC	HDL:TC:TG	LDL	0.97
CAPN3	TG	LDL	0.67
CILP2	TC:TG:LDL	HDL	0.92
GCKR	TG:TC	HDL	0.92
HPR	TC:LDL	HDL	0.6
HNF4A	HDL:TC	TG	0.87
LIPG	HDL:TC	TG	0.84
LDLR	LDL:TC	TG	0.68
SORT1	LDL:TC	TG	0.59

The univariate associations in column 2 are the phenotypes reported as being associated with each SNP in the univariate analyses from [34]. The posterior probability (column 4) shows the assessed probability that the listed trait (column 3) is actually unassociated.

The individual model with greatest estimated probability (38%) was the one with TC, TG and HDL directly associated, and LDL indirectly associated. There followed a long tail of models assigned modest weights; no other individual model had weight 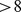
%. This is, perhaps, unexpected: since LDL, HDL and TG are sub-components of TC, one might have expected to see more weight on models in which TC was indirectly associated. We speculate that this result may reflect the fact that in these data, TC, TG and HDL were directly measured, whereas LDL was usually computed from the other measures by the Friedewald formula [35]. If this explanation is correct, then it illustrates the potential for measurement error to complicate the distinction between “direct” and “indirect” associations (see Discussion).

We next applied our framework genome-wide to attempt to identify novel associations. For computational convenience we took a two-stage approach, which first identified approximately 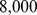 “promising” SNPs by applying simple univariate and multivariate tests to every SNP, and then applied the full Bayesian analysis (using priors 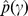 and 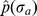 from above) to these promising SNPs. See Detailed Methods for details.

This analysis identified 18 novel independent associations (SNPs more than 0.5Mb apart) with stronger evidence for association in this analysis than the weakest of the 95 associations reported in [34] (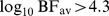). Representative associated SNPs, together with near-by genes, are summarized in Table 2. Consistent with the results above for previously-reported associations, most of these SNPs were assigned high probability of being associated, either directly or indirectly, with all four phenotypes. The one exception was SNP rs17134533 in AKR1C4, which was judged most likely (posterior probability 0.75) to be unassociated with LDL.

**Table 2:** Putative novel associations identified by multivariate analysis of the Global Lipids Data.

snp	chr	pos	MAF	Z_TG_	Z_TC_	Z_LDL_	Z_HDL_	log_10_BF_av_	Gene	Annotation
rs12739698	1	27102620	0.083	3.3	3.4	4.6	−5.1	7.2	NROB2	5' up
rs267733	1	149225460	0.141	−1.0	3.5	5.4	−1.9	4.8	ANXA9	non-syn
rs10490632	2	118295555	0.082	0.8	4.7	5.4	−2.0	5.1	DDX18	intronic
rs13326165	3	52507158	0.207	−4.4	−1.4	−2.1	5.0	4.7	STAB1	intronic
rs762861	4	3411809	0.261	4.5	4.9	4.4	−2.1	5.2	HGFAC/RGS12	5' up/3' down
rs998584	6	43865874	0.491	5.1	0.3	0.0	−4.3	4.5	VEGFA	3' down
rs6951245	7	1024719	0.156	2.0	5.4	3.6	3.3	5.4	C7ORF50	intronic
rs4722551	7	25958351	0.177	3.9	−3.8	−4.9	−1.3	7.5	miR-148a	NA
rs17134533	10	5237098	0.146	−4.9	−3.2	−1.2	−1.8	6.0	AKR1C4	intronic
rs10904908	10	17300296	0.432	−1.6	−4.9	−3.4	−3.6	4.6	VIM	5' up
rs970548	10	45333283	0.246	0.0	−3.8	−2.1	−5.2	5.4	MARCH8	intronic
rs1408579	10	101902184	0.47	2.6	3.2	0.8	3.9	4.8	ERLIN1	intronic
rs11246602	11	51368666	0.126	−0.2	−2.5	−0.7	−5.4	5.3	*	
rs11229252	11	54886216	0.091	0.5	3.4	1.5	5.0	4.9	*	
rs11227638	11	55776161	0.118	−0.6	−2.3	−0.4	−5.0	4.8	*	
rs499974	11	75132669	0.175	−2.1	−2.4	0.3	−4.1	4.4	DGAT2	5' up
rs4942505	13	31861707	0.476	−1.3	−4.5	−5.3	3.1	5.8	BRCA2	intronic
rs10422101	19	57011927	0.265	1.0	−3.6	−2.4	−5.4	5.6	FPR3	intronic

All these SNPs have 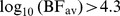, and are more than 0.5Mb from any SNP identified in [34]. (*) These three SNPs map to a region of complex structure on chromosome 11 containing a large number of olfactory receptors, and are in LD with one another despite mapping 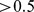 Mb apart (possibly reflecting mapping errors).

One of these associations involves a non-synonymous SNP in ANXA9, which codes for the Annexin-A9 protein. The annexins are a family of calcium-dependent phospholipid-binding proteins, and studies in cows have associated variation in or near ANXA9 with milk-fat yield [36], making this a plausible functional variant that affects lipid levels. More generally, many of the other associations involve SNPs near or in genes that, based on external information, are very plausible candidates for harboring variants that affect lipid levels. For example:

- NROB2 (small heterodimer partner) regulates metabolic pathways, including hepatic bile acid, lipid, and glucose homeostasis [37].
- STAB1 (also known as FEEL-1, and CLEVER-1) codes for the protein stabilin-1 which acts as a scavenger receptor for acetylated low density lipoprotein and oxidized LDL [38]–[40].
- VEGFA codes for the protein “Vascular endothelial growth factor A”, and variants near VEGFA have been implicated in a range of clinical conditions, including diabetic retinopathy [41]–[43] and age-related macular degeneration [44].
- AKR1C4 codes for the enzyme Aldo-keto reductase family 1 member C4, and plays a major role in bile acid biosynthesis [45], which is a major pathway of cholesterol catabolism in mammals.
- VIM codes for Vimentin, which assists in the transport of LDL cholesterol from a lysosome to the site of esterification [46].
- ERLIN1 codes for the protein Erlin-1, which is a member of the prohibitin family of proteins that define lipid-raft-like domains of the endoplasmic reticulum [47]. SNPs near ERLIN1 have previously been associated with plasma levels of alanine-aminotransferase [48], an important liver enzyme.
- DGAT2 encodes one of two enzymes which catalyzes the final reaction in the synthesis of triglycerides, and has been implicated as a major target for the action of niacin in regulating lipids [49], [50].

Thus, although we can not be sure that all the associations in Table 2 reflect true novel associations, both our analyses and these external data suggest that many of them will indeed turn out to be genuine.

## Discussion

We have introduced a framework for association analysis of multiple related phenotypes. This framework unifies such analyses in two ways: it includes standard univariate and standard multivariate tests, as well as many other types of test one might consider, as special cases; and it unifies the problems of *testing* for and *interpreting* associations. Our applications to both real and simulated data suggest that the potential gains in power from multivariate association analyses outweigh the losses in power that can result in specific situations where univariate analyses are most powerful. In addition we emphasized that the settings in which multivariate analyses have increased power can be counter-intuitive: in particular they have power advantages when only one of the phenotypes is associated with genotype, a setting that might naively have been expected to favor the univariate analysis.

Although our framework makes extensive use of Bayesian statistics, we emphasize also its intuitive appeal. In particular, it captures three intuitive principles illustrated in scenarios 1a-c above: i) variables that are unassociated with 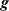 should be controlled for when assessing whether other variables are associated with 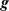 (Figure 1a); ii) variables that are directly associated with 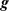 should be treated as a joint multivariate response (Figure 1b); and iii) variables that are only indirectly associated with 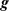 through other *measured* variables provide effectively no additional information for assessing the global null (as in 1c for example). Of course, we do not know *a priori* which of the three categories each variable falls into, and so we try all possibilities, weighting each analysis by how well it explains the observed data. In essence the method conducts an exhaustive search for subsets of variables that are highly associated with 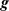 after suitably controlling for other relevant variables, much as a tenacious practitioner might proceed in a more manual analysis. Besides the convenience of being automatic, our Bayesian method also has the advantage that where the “correct” analysis is ambiguous, and many different analyses seem equally consistent with the data, conclusions from these analyses can be combined in a rigorous way. This is important because, with the kinds of subtle effects that are common in genetic association studies, *exactly* which subset of variables are associated with 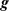 may be impossible to confidently identify; and yet it may be possible to confidently conclude that certain variables are associated with 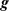, whilst being unsure about others.

### Connections with other work

The field of multivariate statistics is so vast that there are inevitably numerous close connections between our work here and previous work. Here we highlight connections with two particular subfields of multivariate analysis: Directed Acyclic Graphical models (DAGs), and Seemingly Unrelated Regressions (SUR).

#### Connections with Directed Acyclic Graphical Models

Our framework has close connections with Bayesian Directed Acyclic Graphical (DAG) models, particularly for Gaussian data. The main different from typical application of DAGs is that here we focus narrowly on the relationship between one variable (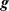) and the remaining variables (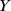), rather than placing equal emphasis on the dependancies and conditional independencies among all variables. This narrower focus helps to simplify both computation and interpretation of results. Furthermore, special properties of 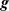 (treatment of 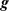 as a randomized intervention) constrains the set of graphical models we consider, disallowing models in which arrows come into 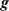.

To establish the connection with DAGs more precisely, note that, for Gaussian data, our null model (equations (13) and (14), with 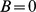) corresponds to a standard Bayesian DAG model for Gaussian 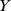
s where the graph connecting the 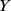 variables is complete: that is, there are edges between all pairs of 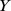 variables, and therefore no conditional independence assumptions are imposed on the 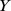 variables. There are many different possible complete DAGs, depending on how one orients the arrows between each pair of variables (the only constraint being that the graph must be acyclic, that is contains no cycles). However, with the inverse Wishart prior, all complete DAGs imply the same probability model [22].

Similarly, the model 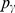 also corresponds to a DAG where the 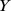 variables are related by a complete graph, but now there are arrows from 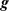 going to each variable in 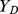, and the directions of the arrows among the 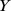 variables are further constrained so that no arrows go from variables in 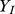 into variables in 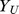 or 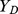, and no arrows go from variables in 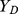 to variables in 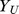. These constraints ensure that the graph is consistent with the factorization (4).

Note that under each model 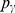 the graph connecting the 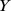
s is complete. To some extent we make this assumption for simplicity: in this particular application the covariance structure of the 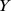
s is a nuisance parameter, about which we care little if at all, and so we are happy not to model it too carefully if we can avoid it. Further, in our current work we are motivated primarily by situations where the dimensionality 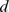 is small compared with 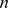, where intuitively there should be plenty of information about the covariance matrix of 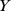, and perhaps little benefit to putting structured priors on this covariance. Indeed, one could argue against more structured models, on the basis that we might want the allocation of variables into groups 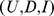 to be driven primarily by the relationship of these variables with 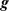, and not with one another. However, in some cases there may be benefits to incorporating structure into the 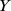
s, particularly if 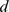 is large (e.g. if the response were expression measures of thousands of genes).

#### Connections with Seemingly Unrelated Regressions

Our work also has close connections with work on Bayesian variable selection in Seemingly Unrelated Regressions (SURs). Seemingly Unrelated Regressions were introduced by [51] as a generalization of standard multivariate regression in which each component of a multivariate outcome may be associated with different explanatory variables. Previous papers that consider Bayesian approaches to selecting explanatory variables associated with each component of the outcome include [52], [53] and, in a genetics context, [2] and [3]. Formally, in the rather special case of *a single explanatory variable*, variable selection in the SUR model is effectively the same as the problem we consider here. However, despite this formal relationship, the aforementioned papers have a very different focus from ours, and – perhaps partly as a result – make use of methods and priors that differ in several details from those presented here. For example, none of them encompasses the concept that some response coordinates may be *indirectly* associated with an explanatory variable; that is, effectively they consider only our categories 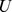 and 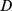, and not 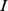. As a result, unlike the framework considered here, they do not include standard univariate analyses as a special case.

Given these connections it is natural to consider extending our framework to simultaneously assess associations with multiple SNPs/covariates. The connection with DAGs might suggest building a single DAG (equivalence class) relating the *Y*s with multiple 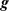
s. However, this approach is unattractive because requiring the 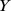
s to have a single DAG structure, shared across all *X*s, would unnecessarily constrain the permitted relationships among variables. For example, it would not allow both 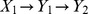 and 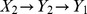 (because the arrows between the 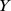 variables are in opposite directions in each case) but this could be a plausible set of relationships (e.g. if there were feedback in the molecular mechanisms relating 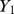 and 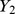). An alternative approach would be to recognize that in our approach each submodel 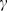 corresponds to a particular prior distribution on 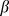 given 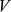, 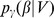, and so an extension to multiple 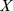 variables could be made by introducing a model indicator 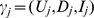 for each variable 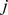, and then assuming that, given 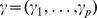 the rows of 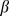 are independent with 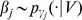. It seems that it should be possible to fit a model along these lines using a Gibbs sampler that at each step updates each row of 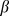 conditional on the other rows, as in [3]. However, in this approach the interpretation of the conditional independencies implied by 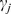 would seem to be complicated by the fact that they would be conditional on the (unknown) values of 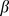 at all SNPs.

### Practical Issues, Critiques, and Areas for further work

We have tried to make our framework as simple and “clean” as possible. Inevitably, when applying it to real data, complications arise, and the veneer of cleanliness starts to be chipped away. For example, in our analysis of the Global Lipids data, the most common partition (TC, TG and HDL being directly associated, LDL being indirectly associated) seems likely to reflect the phenotype measurement protocol rather than interesting biology. Here we attempt to anticipate issues that may arise when applying the framework in practice; in some cases we have partial answers or suggestions for how to approach these issues, but in many cases further work may be required.

#### Beyond multivariate-normal distributions

Perhaps the biggest limitation of our work is that, although the conceptual framework (Section “A unified framework”) is quite general, we provided a practical implementation only for the special case of multivariate normal phenotypes. Furthermore, implementing the framework for other phenotype distributions may prove challenging. One possible approach would be to define 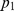 for non-normal phenotypes by applying a link function to a latent multivariate normal distributed variable. For example, one could deal with binary phenotypes 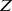 by assuming that 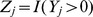, where 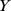 is multivariate normal with 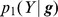 and 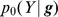 as given here. However, computation of 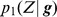, or more generally 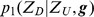, will require the development of efficient approximations to the necessary integrals, which may well not be straightforward.

Another limitation of our work, is that the effect of genotype is assumed to affect only the mean, and not the variances or covariances, of the phenotypes. This assumption also underlies most univariate analysis approaches to genetic association studies, but it is conceivable that some genetic variants (or other variable) could affect phenotype (co)variances instead of, or in addition to, the mean. This represents another potential avenue for future methods development.

A slightly easier, although still potentially tricky, issue, is how to deal with phenotypes that are approximately, but not exactly, multivariate normal. [The methods here actually require only that the likelihood for 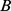 in (13) be approximately multivariate normal, which is weaker than requiring the residuals 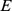 be multivariate normal. Nonetheless, deviations from this assumption remain a potential concern.] In univariate association analyses, we routinely transform quantitative phenotypes to a standard normal distribution (via a rank-quantile transformation), and compute association test statistics or Bayes Factors on the transformed phenotypes. This procedure avoids sensitivity to outlying phenotype values, because it ensures that, under the null, the normal modeling assumptions are met. For multivariate analysis, we recommend this transformation also be applied to quantitative phenotypes before applying the methods here. However, compared with univariate analyses, additional care will still be needed, because transforming each phenotype to be univariate normal does not guarantee that, jointly, the phenotypes are multivariate normal – not even approximately. Furthermore, problems with outliers can be more extreme in multivariate settings. For example, a 20 year-old adult male who is 180 cm tall, and weighs 60 kg, is towards the tails of the distribution in each of height and weight in the USA, but is a much stronger outlier when the two measurements are considered together. This phenomenon can be particularly acute when dealing with strongly correlated phenotypes. Because of this, it may be prudent to check sensitivity of results to the inclusion/exclusion of individuals with the most outlying phenotypes in multivariate space (e.g. those with a large Mahalanobis distances from the mean). Undoubtedly there must be other relevant work on this issue, since robustness to deviations from multivariate normality is relevant also to classical multivariate analyses; however, in a brief literature search we did not find a single widely-adopted solution. In the longer term, a potential alternative to these transformation and outlier-detection based methods would be to modify the normal likelihood assumption to allow for longer tailed distributions such as the 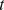 distribution; see, for example, [54] and [55].

#### Choice of coordinate system for 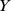


As shown in Corollary 1 above, the Bayes Factor 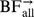, like the standard likelihood ratio test, is invariant to affine transformations of 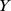. In other words, choice of coordinate system does not affect these simple multivariate tests. In contrast, 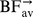, which averages over partitions 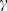, is not so invariant. Indeed, averaging over 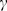 is predicated on the assumption that the coordinate axes have special meaning, since each 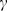 corresponds to an assumption that some coordinates of 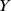 may be associated with 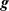 while others are not.

As a result, before computing 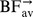, or considering appropriate priors for 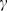, it seems prudent to at least briefly consider choice of appropriate coordinate system. For example, if analyzing *height* and *weight* two possible natural parameterizations would be i) log(*height*) and log(*weight*), or ii) log(*height*) and log(BMI) where 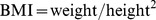. The choice of parameterization affects which models will be naturally included within the partitioning framework outlined here. For example, using i) the framework would include a model where 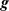 is associated with *height* but not *weight*, whereas ii) would not. One might choose between parameterizations based on this consideration. It would also be possible to consider both parameterizations: because i) and ii) are simply affine transformations of one another the BFs computed from i) and ii) effectively both involve the same null hypothesis in the denominator, and so are directly comparable with one another. This idea was used in [56], in the context of treated and untreated measures of the same phenotype (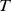 and 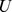): for example, using 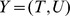 allows for models in which 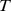 is associated with 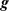 and 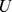 is not, whereas using 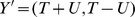 allows for models in which 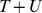 is associated with 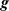 but 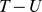 is not (i.e. 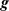 has the same effect on both 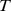 and 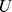). See [56] for more details.

#### Use of PCA for dimension reduction

Another issue related to choice of coordinate system is the possible use of principal components analysis (PCA) on the phenotypes 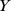 before association analysis. Since the PCs are an affine transformation of 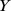, computing 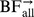 (or a standard multivariate likelihood ratio test) using all the PCs will be equivalent to using the original 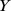. However, computing BFs assessing whether 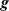 is associated with subsets of the PCs (e.g. just one PC) is different than assessing whether 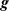 is associated with subsets of the original variables. Whether working with PCs (or combinations of variables obtained by other dimension reduction techniques), is preferable to working with the original variables, will be context specific. In particular, dimension reduction methods may be helpful when analyzing highly structured systems where the phenotypes reflect a small number of underlying physical factors (where small is relative to the number of phenotypes, 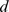). For example, if the components of 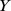 are independent noisy measurements of effectively a single underlying phenotype then the first PC may capture that phenotype effectively, and a univariate test of that PC could be more powerful to detect genotypes associated with that phenotype than a multivariate test of 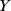. More speculatively, in systems with large numbers of variables, but where the first few PCs capture most of the observed variation, then a multivariate test (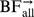) involving the first few PCs may be an effective way to identify associations. However, unless individual PCs are interpretable (which is often not the case; e.g. [57]) considering partitions 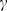 of the PCs may not add much to the analysis. Furthermore, if individual PCs are not easily interpretable then interpreting associations found using PCA may be difficult, especially if examining effect size estimates on the original variables does not yield obvious insights.

#### Interpretation of Partitions, and Latent Factors

**Figure 6:**
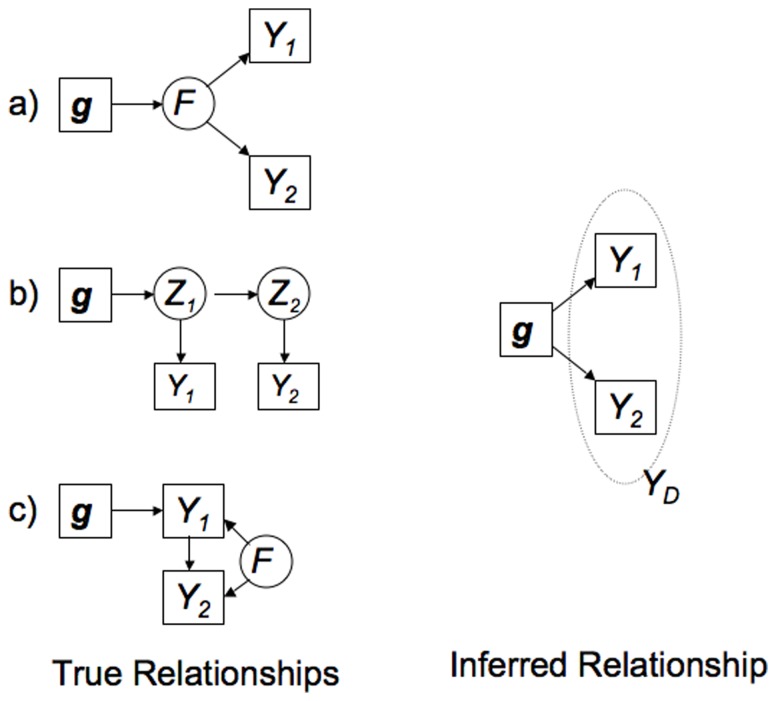
Illustration of potential complications in interpreting direct and indirect effects. The three graphs a)-c) on the left illustrate different hypothetical scenarios that could all lead to the inference that both 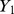 and 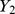 are directly associated with 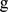 (illustrated by the graph on the right). In each graph square nodes represent observed quantities, and circular nodes represent unobserved quantities. In a) both 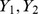 are indirectly associated with 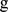 via an unmeasured factor, 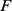. In b) 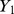 and 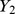 are noisy observations of underlying variables 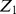 and 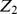, where 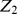 is associated indirectly by  via 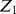. In c) 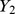 is associated with 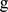 indirectly via 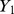, and an unmeasured factor 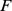 affects both of them. In all three cases both 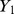 and 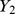 are associated with 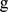, and, further, due to the existence of unmeasured variables, 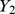 is conditionally dependent on 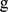 given 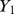, leading to the inference (right) that both 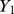 and 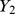 are “directly” associated with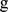.

We have used the terms “directly associated” and “indirectly associated” to refer to variables obeying certain conditional independencies with genotype 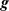. While these terms have value as convenient shorthands that evoke the kinds of relationships between 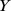 and 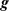 that we might like to infer, we note that in practice there are many reasons to be cautious in interpreting these labels. In particular, it is important to remember that the terms “direct” and “indirect” refer to statistical relationships – specifically, conditional independencies – and not to molecular interactions. Furthermore, conditional dependencies among variables can be affected by the (almost inevitable) presence of unmeasured factors and/or measurement error. Because of this, a variable could be inferred to be “directly” associated with 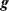, even if in the causal pathway the effect of 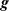 is actually “indirect”. See Figure 6 for examples illustrating some of these issues.

In addition to these interpretational challenges, another difficulty that arises in practice is that data will often be relatively uninformative about which of the three categories 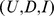 each variable belongs to (even though, in theory, as 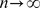, Bayes Factors are consistent for selecting the correct model). For example, it is challenging to find data that are entirely convincing that a particular variable is in 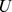: an estimated effect near to 0 is not sufficient, as (with realistic sample sizes) it is difficult to rule out a small non-zero effect. (The same phenomenon occurs in univariate analysis: it is hard to obtain data that strongly favor the null of no association.) For similar reasons it is unusual for data to be strongly informative that an associated variable is in 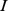: any data that are consistent with the necessary conditional independence, will also be consistent with a small conditional dependence. Another type of inferential difficulty that can arise is if two variables are highly correlated with one another, and both are associated with 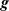: in such cases the data may be consistent, for example, with one variable being in 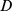 and the other in 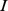, without being informative for which is which.

In contrast to the difficulty of being confident that a variable is in either 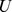 or 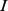, it *is* possible to obtain data that strongly favor a variable being in 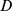: a strong association with 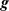, that remains strong when conditioning on other variables, would suffice. Thus, in practice, one tends to see, for each variable, either very strong evidence for being in 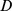, or considerable uncertainty for which category it is in. Of course, these inferential difficulties reflect fundamental limitations of association data, and not limitations of the statistical inference framework.

Finally, a related objection to our framework is that models involving “indirect” associations correspond to very precise conditional independence assumptions that are almost certain to be contravened in any real system. Putting positive weight on these “impossible” models seems counter-intuitive. Similar arguments are sometimes advanced against the use of “point” null hypotheses in settings where the null hypothesis is very unlikely to hold precisely (e.g. when comparing two different drugs, it seems very unlikely that they will have exactly no difference in effect). One response to this criticism is that these models should be viewed as capturing “approximate” conditional independences that exist in the data. However, we admit that, conceptually, it might be more satisfying to attempt to quantify the extent of the conditional dependence, rather than testing whether or not it is equal to 0, as we effectively do here.

Given these difficulties, the reader might be forgiven for wondering whether the distinction between direct associations (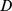) and indirect associations (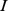) is worth bothering with. We believe it is, if only because including 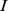 is what makes the framework include the widely-used univariate tests as a special case. Furthermore, if desired one can always focus inference on events that do not make the distinction, such as the event that 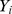 is associated with 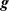, whose probability is 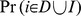 (as in our Global Lipids results, for example).

#### When should variables be analyzed jointly?

A common question put to us about these multivariate methods is whether they are sensitive to the inclusion of additional irrelevant variables. Our response is that additional variables that are *correlated* with the primary variables will seldom be irrelevant in testing any given SNP: either these additional variables will be unassociated with the SNP, and so should be controlled for in testing the primary variables, or they are associated with the SNP in which case it would seem hard to argue that they are irrelevant.

Of course, if variables are both approximately uncorrelated with one another, and also judged unlikely to share any common genetic effects, then there is little to be gained in treating them jointly. Further, the larger the number of variables included in the analysis, the more careful one might need to be about specifying priors on 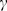 and also 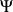, and so variables should not be included willy-nilly.

#### Formalizing a hierarchical model

Our analysis of the Global Lipids data took an informal Empirical-Bayes-like approach to setting the prior distributions 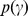 and 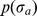. In particular, this informal approach used a two stage process, first identifying the strongest associations in the data, and then used these to estimate 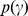 and 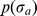. Certainly this informal approach could be improved upon. The most obvious improvement would be to attempt to build a formal hierarchical model, and then estimate 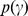 and 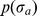, as well as the proportion of nulls 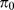, from all the data. However, this approach is not without its challenges, including the challenge of properly coping with correlations among SNPs (linkage disequilibrium). Perhaps a more fundamental issue is whether it might make more sense to build the hierarchical model at the level of the effect sizes (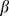), rather than at the level of the partitions/models (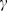). The point here is that our framework, when implemented into a hierarchical model, encourages the lumping together of SNPs with very different effects. For example, in our example of associating SNPs with 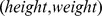, the class of SNPs that have a direct effect on both could include SNPs where one allele increases both 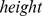 and 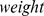, as well as SNPs where one allele decreases 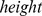 but increases 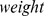. These SNPs seem qualitatively different, and, in the context of hierarchal modeling, treating them as coming from the same “model” seems unnatural. We see the development of hierarchical modeling methods at the level of the effect sizes 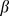 as a potentially interesting area for future work.

### Detailed Methods (Global Lipids Analysis)

We downloaded the tables of summary results of the large meta-analysis of Triglycerides, Total Cholesterol, LDL-C and HDL-C (TC2010.zip, HDL2010.zip, LDL2010.zip, TG2010.zip) from http://www.sph.umich.edu/csg/abecasis/public/lipids2010/. These tables include a combined 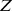 statistic (computed from all available individuals contributing to the meta-analysis at that SNP, and corrected for population stratification by Genomic Control [58]), 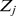, and a sample size 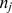 for each SNP 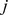. We also obtained a file of additional information on each SNP from X. Wen (personal communication) that included an estimate of the minor allele frequency for each SNP (from the studies that contributed to the meta-analysis when available; using data from the 1000G project when this was not available). Because of different protocols in each study, the 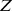 statistics are based on different sample sizes, for each SNP and each phenotype. We defined a single sample size, 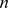, for each SNP, as the minimum of these sample sizes across the four phenotypes. We excluded from our analysis SNPs with 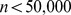.

The Bayes Factors depend on the matrix of phenotype correlations, 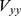. Since we did not have access to the phenotype correlations in each study (and in any case, the analysis would be complicated by the fact that these correlations may differ across studies) we took the following approach to obtain an approximate value for 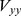. Under the null hypothesis, the correlation of the 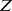 statistics, 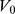, is equal to the correlation of the phenotypes 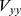. To approximate 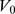 (and hence 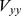) we first identified a set of putative null SNPs, by taking all SNPs with 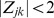 for all four phenotypes 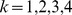. Let 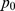 denote the number of such SNPs and 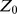 denote the resulting 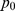 by 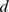 matrix. We estimated 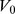 by the 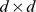 correlation matrix, 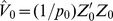.

In our genome scan, to reduce computation, we first used simple multivariate and univariate tests to identify a set of “promising” SNPs on which to perform a full Bayesian association analysis. Specifically, for each SNP 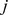 we computed a multivariate test statistic, 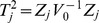, and a corresponding 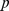 value based on the assumption that, under the null, 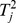 will have a chi-squared distribution on 4 degrees of freedom (df). We also computed a univariate 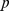 value for each trait, by comparing 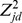 with a chi-squared distribution on 1 df. We marked a SNP as “promising” if any of its univariate 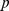 values, or its multivariate 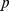 value was 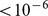. In total 8 065 SNPs met this criteria.

Next, for all 8 065 promising SNPs, we computed Bayes Factors for all partitions 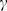. In Supplementary Information S1 (Section S.1) we give an algorithm for computing the Bayes Factors using the summary statistic matrices 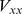, 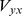 and 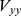. To apply this algorithm to these data, where we do not have direct access to these matrices, we approximated these matrices from the 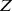 scores, sample size 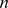, and minor allele frequency 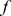 as follows. 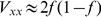
(33)

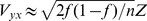
(34)

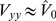
(35)


The first of these comes from the fact that 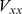 is a vector of genotype variances, and the expected genotype variance, under Hardy Weinberg equilibrium, is 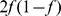. The second of these comes from the fact that 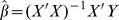 and 
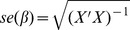
 so 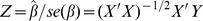 or, rearranging, 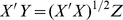.

We took an “Empirical Bayes” approach to obtain joint prior probabilities for the partition 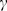 and effect size variance 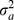 for associated SNPs. Specifically, for each of the 95 SNPs reported as being associated with lipids in [34] we computed 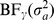 for all 66 possible values of 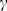, and on a discrete grid of values for 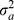, 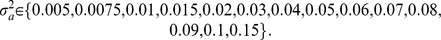


We then estimated the prior probability of each combination, 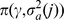, by maximizing the likelihood 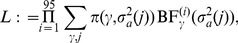
(36)where 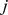 indexes the different grid values for 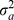 and 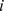 indexes the 95 SNPs. We maximized the likelihood using an EM algorithm; four independent runs of the algorithm from different starting points produced essentially identical results. Because the 95 SNPs used in this estimation procedure are biased towards the most strongly associated SNPs, we expect the resulting estimates of 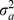 to be biased upwards. However, this simple data-driven approach seemed preferable to simply picking values for these prior parameters more arbitrarily.

The associations reported in Table 2 were identified by first ranking SNPs by their Bayes Factor for association (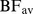), and then removing multiple associations that were likely due to LD by eliminating any SNP that was within 0.5Mb of a higher-ranked SNP. We also removed a SNP (rs2746150) on chromosome 6, which we judged likely a secondary association due to LD with previously-reported associations in the MHC region. We annotated the remaining SNPs with respect to near-by genes using SNPnexus http://www.snp-nexus.org/ [59], [60] and by manual inspection in the ENSEMBL browser www.ensembl.org.

## Supporting Information

Supplementary Information S1 (PDF)
