# Correction: A Unified Framework for Association Analysis with Multiple Related Phenotypes

**DOI:** 10.1371/journal.pone.0213951

**Published:** 2019-03-19

**Authors:** Matthew Stephens

In the Specifying *p*_*γ*_ subsection of the Methods, there is an error in Equation (4). Please view the complete, correct equation here:

$$p_{\gamma }(Y|g)=p_{0}(Y_{U})p_{1}(Y_{D}|Y_{U},g)p_{0}(Y_{I}|Y_{U},Y_{D}).$$

In the Multivariate normal phenotypes subsection of the Methods, there is also an error in Equation (26). Please view the complete, correct equation here:

$$Y_{D}|\mu ,\alpha ,V,\beta =\mu +Y_{U}\alpha +g\beta +E$$
