# Supplementary material for: A Unified Framework for Association Analysis with Multiple Related Phenotypes
Source: PLoS One. 2013 Jul 5;8(7):e65245. doi: 10.1371/journal.pone.0065245 (PMC3702528; doi:10.1371/journal.pone.0065245)
Supplement: Supplementary Information S1 — (PDF) [file pone.0065245.s001.pdf]

Supplementary Information S1 for "A unified framework for association analysis with multiple related phenotypes"

Matthew Stephens

S.1 Efficient Algorithm for computing BFs for all SNPs

The following algorithm takes advantage of rank-one matrix updates, and the fact that Bayes factors for each partition depend only on summary statistics, to avoid computing things multiple times for each SNP. (Note that further improvements in efficiency could be made; for example by reusing computations across partitions that share Y_U in common, but this seems unnecessary at this point.)

Algorithm S.1.1. *Consider computing the single SNP Bayes Factors (7) for multivariate outcomes $Y(n \times d)$ and p SNPs with genotypes $X(n \times p)$. Assume that the columns of both Y and X have been centered on zero by subtracting the column means. Take the summary statistic matrices*

$$V_{yx} = (1/n)Y'X \qquad (d \times p) \qquad \text{(S.1.1)}$$
$$V_{yy} = (1/n)Y'Y \qquad (d \times d) \qquad \text{(S.1.2)}$$

and let V_{xx} be a p-vector containing the variances of the columns of X, $V_{xx}[j] = (1/n)||X_{\cdot j}||_2$ where $||x||_2 := \sum_i x_i^2$.

For any matrix V we will use the notation $V[U, D]$ to denote the submatrix of V consisting of the rows with indices in the set U and columns with indices in the set D.

For a given partition $\gamma = (U, D, I)$ we can compute the Bayes factor BF_γ for all p SNPs as follows:

1. *Compute the Cholesky decomposition L_{UU} of the submatrix $V_{yy}[U, U]$. (So $L'_{UU}L_{UU} = V_{yy}[U, U]$.)*

2. *Solve $L_{UU}b = V_{yy}[U, D]$ and $L_{UU}c = V_{yx}[U,]$ for matrices $b(d_U \times d_D)$ and $c(d_U \times p)$. (If $d_U = 0$ then set $b = c = 0$ in what follows.)*

3. *Set $C_j = V_{xx}[j] - ||c_{\cdot j}||_2$.*

4. *Set $u = V_{yx}[D,] - b'c$ a $d_D \times p$ matrix.*

5. *Compute the Cholesky decomposition L_0 for $V_0 = V_{yy}[D, D] - b'b$.*

6. *Solve $L_0 a = u$ for a a $d_D \times p$ matrix.*

7. *Set* $\lambda_j = \sigma_a^{-2}/n_j C_j$ *and* $k_j = 1/(1+\lambda_j)$.

8. *For the* j*th SNP return* $BF_j = 0.5 d_D \log(1-k_j) - 0.5(n_j + m - d_I)\log(1-(k_j/C_j)||a_{.j}||_2)$.

The following R code implements this algorithm. (Note that for convenience the loop over partitions includes partitions with $|D| = 0$; these partitions are assigned 0 prior probability, and their Bayes Factors are set to be 1.)

```
computeprior = function(z,pi){
  dvec = tabulate(z+1,nbin=3)
  d = length(z)
  return(ifelse(dvec[2]>0,(1-pi)*(1/d) * (1/(dvec[2]+dvec[3])) *
          1/choose(d, dvec[1]) * 1/choose(d-dvec[1],dvec[2]), 0))

}

#picks out the partition gamma with all 1s (ie all in D)
allones = function(gamma){return(prod(gamma==1))}

#note the "drop=FALSE" commands below stop R collapsing matrices into vectors inappropriately
#VYX \approx (1/n) Y'X is d by p
#VYY \approx (1/n) Y'Y is d by d
#VXX is a p-vector of the estimated variances of the SNP
logBF.fromVSummaries = function(VYX,VYY,VXX,U,D,n,m,d,sigmaa){

dd = sum(D)
du= sum(U)
p = dim(VYX)[2]

if(du>0){
LUU = chol(VYY[U,U,drop=FALSE]) # a du by du matrix
VUD = VYY[U,D,drop=FALSE]      #a du by dd matrix of the correlations of Yu with Yd

c = cbind(forwardsolve(t(LUU),VYX[U,,drop=FALSE]))#c solves LUU'c = phiU, c is a du by p matrix
b = cbind(forwardsolve(t(LUU), VUD))  # b is du by dd, and solves LUU' b = VUD,
#so b'b = VUD' LUU^-1 LUU'^-1 VUD = VUD' (LUU'LUU)^-1 VUD = VUD'VYYU^-1 VUD
} else{c=matrix(0,nrow=1,ncol=p); b=matrix(0,nrow=1,ncol=dd);}

C = VXX - colSums(c*c)

u = VYX[D,,drop=FALSE] - crossprod(b,c)

V0 = VYY[D,D,drop=FALSE] - crossprod(b)
L0 = chol(V0)
a = forwardsolve(t(L0),u)
lambda = sigmaa^(-2)  / (n*C)
k = as.numeric(1/(1+lambda))
return((dd/2) * log(1-k) - 0.5*(n+m-(d-sum(D)-sum(U)))*log(1-(k/C) *colSums(a*a)))
}
```

```
#G is an n by p matrix of SNP genotypes
#Y is an n by d matrix of phenotypes
#single-SNP BFs are computed for each SNP (column of G)
logBF.rankone.matrix = function(G,Y,sigmaa,pi0=0.5,m=0){
if(is.null(dim(G))){G=cbind(G)} # turn a vector of genotypes into a matrix
subset = complete.cases(Y) & complete.cases(G)
Y=Y[subset,,drop=FALSE]
G=G[subset,,drop=FALSE]
n = dim(Y)[1]
d = dim(Y)[2]
p = dim(G)[2] #number of SNPs
if(m==0){m = d-1}

Y =scale(Y,center=T,scale=F)  #center Y and G to avoid needing intercept in regression
G = scale(G,center=T,scale=F)

VYX = (1/n)*crossprod(Y,G) # this is (1/n) t(Y) %*% G, a d by p matrix
VYY = (1/n)*crossprod(Y) # (1/n) t(Y) %*% Y, a d by d matrix
VXX = (1/n)*colSums(G*G) # a p vector of (1/n) ||g|| values

prior = rep(0,3^d)
gamma=matrix(0,nrow=3^d,ncol=d)
lbf = matrix(0,nrow=3^d, ncol=p)

for(i in 0:(3^d-1)){
  for(j in 1:d){
    gamma[i+1,j]= (i %% 3^j) %/% 3^{j-1}
  }
  prior[i+1] = computeprior(gamma[i+1,],pi0)
  U = (gamma[i+1,]==0)
  D = (gamma[i+1,]==1)
  if(prior[i+1]>0){
    BF = 0
    for(ss in 1:length(sigmaa)){
      BF = BF+exp(logBF.fromVsummaries(VYX,VYY,VXX,U,D,n,m,d,sigmaa[ss]))
      #note we just don't bother computing for models with prior = 0
    }
    lbf[i+1,]=log(BF/length(sigmaa))
  } else {lbf[i+1,] = 0}
}
prior[1] = pi0

BF=exp(lbf)
posterior = prior[-1]*BF[-1,,drop=FALSE]
normalize=function(x){return(x/sum(x))}
posterior = apply(posterior,2,normalize)
p0=t(gamma[-1,]==0) %*% posterior
p1=t(gamma[-1,]==1) %*% posterior
```

```
p2=t(gamma[-1,]==2) %*% posterior

#divide by log(10) to convert everything to log base 10
lbfav = log(colSums(prior[-1]*exp(lbf[-1,,drop=FALSE]))/sum(prior[-1]))/log(10)
lbfuni.comps = apply(lbf[rowSums(gamma)==(2*d-1),,drop=FALSE],2,rev)/log(10)
lbfuni = log10(apply(10^lbfuni.comps,2,mean))
lbfall = lbf[which.max(apply(gamma,1,allones)),,drop=FALSE]/log(10)
lbf = lbf/log(10)
return(list(prior=prior,gamma=gamma,lbf=lbf,lbfav = lbfav,lbfuni=lbfuni,
lbfall=lbfall,lbfuni.comps=lbfuni.comps,p0=p0,p1=p1,p2=p2))
}
```

The algorithm is based on the following Lemma:

Lemma S.1.1. *Consider the Bayes Factor comparing the models*

$$Y \sim BMVR(X = [X_0, X_1]; K, \Psi, m) \tag{S.1.3}$$

$$Y \sim BMVR(X_0; K_0, \Psi, m) \tag{S.1.4}$$

where Y is an $n \times d$ matrix; X_0 and X_1 are $n \times p_0$ and $n \times p_1$ matrices; K_0 is a $p_0 \times p_0$ matrix; and

$$K = \begin{pmatrix} K_0 & 0 \\ 0 & K_1 \end{pmatrix} \tag{S.1.5}$$

where K_1 is $p_1 \times p_1$.

Assume that $K_1 = \lambda X_{1\perp 0}' X_{1\perp 0}$ for some scalar λ where $X_{1\perp 0}$ is the part of X_1 that is orthogonal to X_0: $X_{1\perp 0} := (I_n - P_0)X_1$, where $P_0 := X_0(X_0'X_0)^{-1}X_0'$ is the projection matrix that projects onto the column space of X_0. Then in the limit $K_0 \to 0$ and $\Psi \to 0$, assuming that relevant matrices are invertible, this Bayes Factor is given by

$$BF^{\rightarrow} = [1-k]^{p_1 d/2} \left| I_{p_1} - k(X_{1\perp 0}' X_{1\perp 0})^{-1} u' \mathrm{RSS}_0^{-1} u \right|^{-(n+m)/2} \tag{S.1.6}$$

where $k = (1+\lambda)^{-1}$, $u = Y'X_{1\perp 0}$ is a $d \times p_1$ matrix, and $\mathrm{RSS}_0 = Y'(I - P_0)Y$ is the standard residual sums of squares matrix from regressing Y on X_0.

Note S.1.1.

1. *In the case $X_1 = \boldsymbol{g}$ that we consider here, the dimension p_1 is 1, and so the determinant is trivial to compute. Further, in this case $X_{1\perp 0}' X_{1\perp 0}$ is a scalar, so the condition on K_1 always holds for some λ: that is, the expression for $BF^{\rightarrow}$ holds for any K_1, with $\lambda = K_1 / X_{1\perp 0}' X_{1\perp 0}$.*

2. *This lemma allows the Bayes Factor to be computed efficiently for a large number of SNPs (X_1), and a given Y and X_0. Note, in particular, that the matrices RSS_0^{-1} and P_0 do not depend on X_1, and so any relatively expensive calculations for these matrices (e.g. Cholesky decompositions) need be performed only once for all SNPs. Furthermore, quantities whose computation increases with n, such as $Y'Y$, need only be computed once for all partitions γ.*

3. *(Connection with Approximate Bayes Factors.) Consider the case* $X_1 = \boldsymbol{g}$ *and* $X_0 = \mathbf{1}$*. Then* $X_{1\perp 0} = \tilde{\boldsymbol{g}}$*, the vector of mean-centered genotypes (centered to have mean 0). Note that* $BF^{\rightarrow}$ *is invariant to rescaling of* Y*, so without loss of generality assume that each component of* Y *has variance 1. Then* $Z := (X'_{1\perp 0}X_{1\perp 0})^{-0.5}u$ *is a vector of* Z*-scores, and* $\hat{V}_{yy} := (1/n)\mathrm{RSS}_0$ *is the correlation matrix of the phenotypes* Y*, which is also the correlation matrix of the* Z*-scores under* H_0*. Then*

$$BF^{\rightarrow} = (1-k)^{d/2}(1-(k/n)T^2)^{-(n+m)/2} \tag{S.1.7}$$

where $T^2 := Z'\hat{V}_{yy}^{-1}Z$ *is a multivariate test statistic for* H_0*, which has a chi-square distribution with* d *degrees of freedom under* H_0*.*

Further, using $\lim_{n\to\infty}(1-x/n)^{-(n+m)/2} = \exp(x/2)$*, for large* n *we have*

$$BF^{\rightarrow} \approx (1-k)^{d/2}\exp[(k/2)T^2]. \tag{S.1.8}$$

This is the analogue of the approximate Bayes Factors in Wakefield (2009) and Johnson (2008).

A proof of Lemma S.1.1 follows some definitions and Lemma S.1.2.

Definition S.1.1. *Recalling that*

$$\mathrm{RSS}(Y|X,K) := Y'Y - Y'X(X'X+K)^{-1}X'Y,$$

we define

$$\Delta\mathrm{SS}(Y|X,K) := Y'X(X'X+K)^{-1}X'Y.$$

Note that for $K = \mathbf{0}$*, a matrix with all 0 entries,* $\mathrm{RSS}(Y|X,\mathbf{0})$ *is the usual residual sums of squares matrix from regressing* Y *on* X*, and* $\Delta\mathrm{SS}(Y|X,\mathbf{0})$ *is the change in the sums of squares matrix due to regression on* X*. For non-zero* K*, we will refer to* $\mathrm{RSS}(Y|X,K)$ *and* $\Delta\mathrm{SS}(Y|X,K)$ *as Bayesian analogues of these quantities (although the analogy is imperfect).*

Definition S.1.2. *Given matrices* X_0 *and* X_1*, each with* n *rows, we define* $X_{1\perp 0}$ *to be the part of* X_1 *that is orthogonal to* X_0*; that is,*

$$X_{1\perp 0} := (I_n - P_0)X_1,$$

where $P_0 := X_0(X_0'X_0)^{-1}X_0'$ *is the projection matrix that projects onto the column space of* X_0*.*

Lemma S.1.2. *a) The change in sums of squares due to* $X = [X_0, X_1]$ *can be decomposed as*

$$\Delta\mathrm{SS}(Y|[X_0,X_1],\mathbf{0}) = \Delta\mathrm{SS}(Y|X_0,\mathbf{0}) + \Delta\mathrm{SS}(Y|X_{1\perp 0},\mathbf{0}).$$

b) The Bayesian analog of $\Delta\mathrm{SS}$ *is related the* $\Delta\mathrm{SS}$ *of an OLS regression by:*

$$\Delta\mathrm{SS}(Y|X,K) = \Delta\mathrm{SS}(\tilde{Y}|\tilde{X},\mathbf{0})$$

where $\tilde{Y} = \begin{pmatrix} Y \\ 0 \end{pmatrix}$ *and* $\tilde{X} = \begin{pmatrix} X \\ L \end{pmatrix}$*, with* L *being any matrix such that* $L'L = K$*. [Note: in effect, this means that Bayesian regression can be performed by adding "pseudo-observations" of 0 to* Y*, and extending the design matrix* X *by* L*.]*

c) *If $K = \lambda X'X$ then the Bayesian analog of ΔSS is a shrunken version of ΔSS from OLS:*

$$\Delta\text{SS}(Y|X, K) = (1+\lambda)^{-1}\Delta\text{SS}(Y|X, \mathbf{0}).$$

d) *If $K = \begin{pmatrix} 0 & 0 \\ 0 & K_1 \end{pmatrix}$ then*

$$\Delta\text{SS}(Y|X, K) = \Delta\text{SS}(Y|X_0, \mathbf{0}) + \Delta\text{SS}(Y|X_{1\perp 0}, K_1).$$

e) *Following on from d), if $K_1 = \lambda X'_{1\perp 0}X_{1\perp 0}$ then*

$$\Delta\text{SS}(Y|X, K) = \Delta\text{SS}(Y|X_0, \mathbf{0}) + k\Delta\text{SS}(Y|X_{1\perp 0}, \mathbf{0}).$$

where $k = (1+\lambda)^{-1}$.

f) *Following on from e),*

$$\text{RSS}(Y|X, K) = \text{RSS}(Y|X_0, \mathbf{0}) - k\Delta\text{SS}(Y|X_{1\perp 0}, \mathbf{0}).$$

Proof. a) Let $C(X)$ denote the column space of X, and P denote the projection matrix into $C(X)$: $P = X(X'X)^{-1}X'$. Note that, because $X_{1\perp 0}$ is the part of X_1 that is orthogonal to X_0, $C([X_0, X_1]) = C([X_0, X_{1\perp 0}])$. Further, since $C(X_0)$ and $C(X_{1\perp 0})$ are orthogonal, projection onto $C(X_0, X_{1\perp 0})$ can be decomposed into orthogonal parts, the projection into $C(X_0)$ and the projection into $C(X_{1\perp 0})$. That is $P = P_0 + P_{1\perp 0}$. Thus $Y'PY = Y'P_0Y + Y'P_{1\perp 0}Y$, and the result follows.

b) This follows directly from the definition of ΔSS on noting that $\tilde{X}'\tilde{X} = X'X + K$.

c) This follows directly from substituting $K = \lambda X'X$ for K in the definition of ΔSS.

d) Let $X = [X_0, X_1]$ and L_1 be any matrix such that $L_1'L_1 = K_1$. Also let $\tilde{Y} = \begin{pmatrix} Y \\ 0 \end{pmatrix}$. Then

$$\begin{aligned}
\Delta\text{SS}(Y|X, K) &= \Delta\text{SS}(\tilde{Y}|\begin{pmatrix} X_0 & X_1 \\ 0 & L_1 \end{pmatrix}, \mathbf{0}) \quad \text{[by b)]} \\
&= \Delta\text{SS}(\tilde{Y}|\begin{pmatrix} X_0 \\ 0 \end{pmatrix}, \mathbf{0}) + \Delta\text{SS}(\tilde{Y}|\begin{pmatrix} X_{1\perp 0} \\ L_1 \end{pmatrix}, \mathbf{0}) \quad \text{[by a)]} \\
&= \Delta\text{SS}(Y|X_0, \mathbf{0}) + \Delta\text{SS}(Y|X_{1\perp 0}, K_1) \quad \text{[by b)]}
\end{aligned}$$

e) This follows by applying *c)* to the term $\Delta\text{SS}(Y|X_{1\perp 0}, K_1)$ in *d)*.

f) This follows directly from *e)* from the definitions of RSS and ΔSS.

□

Proof of Lemma S.1.1.

Proof. From (16) the Bayes factor is given by

$$\left[\frac{|K|}{|K_0|}\frac{|X_0'X_0+K_0|}{|X'X+K|}\right]^{d/2}\left[\frac{|\mathrm{RSS}(Y|X_0,K_0)+\Psi|}{|\mathrm{RSS}(Y|X,K)+\Psi|}\right]^{(n+m)/2}. \tag{S.1.9}$$

Note that $|K|/|K_0| = |K_1|$. Also, in the limit $K_0 \to 0$ we have

$$X'X+K = \begin{pmatrix} X_0'X_0 & X_0'X_1 \\ X_1'X_0 & X_1'X_1+K_1 \end{pmatrix}. \tag{S.1.10}$$

If $X_0'X_0$ is invertible then the determinant of this matrix is, by standard properties of the determinant, given by

$$\begin{aligned} |X'X+K| &= |X_0'X_0||X_1'X_1+K_1-X_1'X_0(X_0'X_0)^{-1}X_0'X_1| & \text{(S.1.11)}\\ &= |X_0'X_0|(1+\lambda)^{p_1}|X_1'(I_n-P_0)X_1| & \text{(S.1.12)}\\ &= |X_0'X_0|[(1+\lambda)/\lambda]^{p_1}|K_1| & \text{(S.1.13)}\end{aligned}$$

where the second line is obtained by substituting $\lambda X_1'(I-P_0)X_1$ for K_1. Thus the first term in (S.1.9) is given by $[\lambda/(1+\lambda)]^{p_1 d/2} \equiv [1-k]^{p_1 d/2}$

Turning now to the second term, in the limit $K_0 \to \mathbf{0}$ and $\Psi \to \mathbf{0}$ the ratio of the determinants becomes $|\mathrm{RSS}(Y|X_0,\mathbf{0})|/|\mathrm{RSS}(Y|X,K)|$. By Lemma S.1.2f) the denominator is

$$\begin{aligned} \mathrm{RSS}(Y|X,K) &= \mathrm{RSS}(Y|X_0,\mathbf{0}) - kY'X_{1\perp 0}(X_{1\perp 0}'X_{1\perp 0})^{-1}X_{1\perp 0}'Y & \text{(S.1.14)}\\ &= \mathrm{RSS}(Y|X_0,\mathbf{0}) - kAB & \text{(S.1.15)}\end{aligned}$$

where $A := Y'X_{1\perp 0}$ is $d \times p_1$ and $B := (X_{1\perp 0}'X_{1\perp 0})^{-1}X_{1\perp 0}'Y'$ is $p_1 \times d$. Then, by standard properties of the determinant (e.g. Wikipedia),

$$|\mathrm{RSS}(Y|X,K)| = |\mathrm{RSS}(Y|X_0,\mathbf{0})||I_{p_1} - kB\mathrm{RSS}(Y|X_0,\mathbf{0})^{-1}A| \tag{S.1.16}$$

and the result follows. □

S.2 Proof of Proposition 1

To prove this proposition we use the following Lemma in conjunction with Lemma S.1.1 above.

Lemma S.2.1. *Let $\Lambda(Y \sim X_1|X_0)$ denote the usual likelihood ratio test statistic for testing whether Y is associated with X_1 given X_0 in a multivariate normal regression model. That is, $\Lambda(Y \sim X_1|X_0)$ is the likelihood ratio test of $B_1 = 0$ in*

$$Y = X_0B_0 + X_1B_1 + E, \tag{S.2.1}$$

where Y, E are $n \times d$, X_0 is $n \times p_0$ and X_1 is $n \times p_1$, B_0 is $p_0 \times d$ and B_1 is $p_1 \times d$, and the rows of E are independent and identically distributed d-variate normal with unknown covariance matrix V. Then

$$\Lambda(Y \sim X_1|X_0) = \left|I_{p_1} - (X_{1\perp 0}'X_{1\perp 0})^{-1}u'\mathrm{RSS}_0^{-1}u\right|^{n/2} \tag{S.2.2}$$

where $X_{1\perp 0}$, u and RSS_0 are as in Lemma S.1.1.

Proof. By definition, the likelihood ratio test statistic is the ratio of determinants

$$\Lambda(Y \sim X_1|X_0) = \left[\frac{|\mathrm{RSS}(Y|[X_0, X_1], \mathbf{0})|}{|\mathrm{RSS}(Y|X_0, \mathbf{0})|}\right]^{n/2}. \tag{S.2.3}$$

Setting $\lambda = 0$ (so $k = 1$ and $K = \mathbf{0}$) in (S.1.16) in the proof above we have

$$|\mathrm{RSS}(Y|[X_0, X_1], \mathbf{0})| = |\mathrm{RSS}(Y|X_0, \mathbf{0})||I_{p_1} - B\mathrm{RSS}_0^{-1}A)|, \tag{S.2.4}$$

and the result follows. □

We can now prove Proposition 1 which we restate here for convenience.

Proposition 1. *The Bayes Factor* $BF_\gamma^{\rightarrow}$ *is related to the likelihood ratio statistic* $\Lambda_\gamma := \Lambda(Y_D \sim \boldsymbol{g}|Y_U)$ *by*

$$BF_\gamma^{\rightarrow} = (1-k)^{|D|/2}(k\Lambda_\gamma^{2/n} + 1 - k)^{(n+m-d+|U|+|D|)/2} \tag{S.2.5}$$

with $k = 1/(1 + (\sigma_a^2 \tilde{\boldsymbol{g}}'\tilde{\boldsymbol{g}})^{-1})$*, where* $\tilde{\boldsymbol{g}}$ *denotes the vector of residuals from OLS regression of* $\boldsymbol{g}$ *on* Y_U *(including an intercept).*

Proof. Recall that $\mathrm{BF}_\gamma^{\rightarrow}$ is the Bayes Factor for comparing two BMVRs, (22) with (23). Therefore we can apply Lemma S.1.1 with $Y = Y_D, X_0 = [\mathbf{1}, Y_U], X_1 = \boldsymbol{g}, K_1 = \sigma_a^{-2}, m = m - d + |U| + |D|$ to obtain

$$\mathrm{BF}_\gamma^{\rightarrow} = (1-k)^{|D|/2} \left|1 - k(X_{1\perp 0}'X_{1\perp 0})^{-1}u'\mathrm{RSS}_0^{-1}u\right|^{-(n+m-d+|U|+|D|)/2}. \tag{S.2.6}$$

It then remains to show that $(X_{1\perp 0}'X_{1\perp 0})^{-1}u'\mathrm{RSS}_0^{-1}u = 1 - \Lambda_\gamma^{2/n}$. However, this follows directly from Lemma S.2.1. □

S.3 Simulation Code

The following code was used to generate the six different simulation scenarios described in the text.

```
if(type==1){ #Multivariate 1
Y[1,] =Z[1,]+a*X
Y[2,] =(Z[2,]+Z[1,])/sqrt(2)
Y[3,]=Z[3,]+a*X
Y[4,]=(Z[4,]+Z[2,]+Z[3,])/sqrt(3)
Y[5,]=(Y[3,]+Z[5,])/sqrt(2)
}
if(type == 2){ #Independence
Y[1,] =Z[1,]+a*X
Y[2,] =Z[2,]
Y[3,]=Z[3,]
Y[4,]=Z[4,]
```

```
Y[5,]= Z[5,]
}
if(type==3){ #Rest indirectly associated
Y[1,] =Z[1,]+a*X
Y[2,] =(Z[2,]+Y[1,])/sqrt(2)
Y[3,]=(Z[3,]+0.5*Y[1,])/sqrt(1+0.5^2)
Y[4,]=(Z[4,]+0.2*Y[1,])/sqrt(1+0.2^2)
Y[5,]= (Z[5,]+0.1*Y[1,])/sqrt(1+0.1^2)
}
if(type==4){ # Latent factor
f = a*X + 0.5*rnorm(n)
Y[1,] = 0.5*Z[1,] + 0.3*f
Y[2,]= 0.5*Z[2,] + 0.2*f
Y[3,] = 0.5*Z[3,] - 0.3*f
Y[4,] = 0.5*Z[4,] + 0.5*f
Y[5,] = 0.5*Z[5,]+ 0.2*f
}
if(type==5){ # Multivariate 2
Y[1,] = Z[1,]
Y[2,]=  Z[2,]
Y[3,] = Z[3,]
Y[4,] = (Z[4,] + Y[1,] +Y[2,])/sqrt(3)+ a*X
Y[5,] = (Z[5,] + Y[3,])/sqrt(2) + a*X
}
if(type==6){ # Rest unassociated
Y[1,] = Z[1,]
Y[2,]=  Z[2,]
Y[3,] = Z[3,]
Y[4,] = Z[4,]
Y[5,] = (Z[5,] + Y[1,] + Y[2,] + Y[3,] + Y[4,])/sqrt(5) + a*X
}
```

S.4 Partition property of BMVR

The following partition property is used to obtain the expressions (22) and (23). It is not new, but is included for completeness.

Proposition S.4.1 (Partition Property of BMVR)**.** *Suppose $Y \sim BMVR(X; K, \Psi, m)$ with Ψ diagonal. Now consider partitioning Y and Ψ into groups of d_1, d_2 variables*

$$Y = (Y_1, Y_2) \qquad \Psi = \begin{pmatrix} \Psi_{11} & 0 \\ 0 & \Psi_{22} \end{pmatrix}. \tag{S.4.1}$$

Then

$$Y_1 \sim BMVR(X; K, \Psi_{11}, m - d_2) \tag{S.4.2}$$
$$Y_2|Y_1 \sim BMVR([X, Y_1]; \tilde{K}, \Psi_{22}, m) \tag{S.4.3}$$

where

$$\tilde{K} = \begin{pmatrix} K & 0 \\ 0 & \Psi_{11} \end{pmatrix}. \tag{S.4.4}$$

This partition property says that a Bayesian multivariate regression of Y on X, with priors given by (14) and (15), and with Ψ diagonal, is equivalent to a Bayesian multivariate regression of Y_1 on X, followed by a Bayesian multivariate regression of Y_2 on X and Y_1. Note that while in non-Bayesian settings it is well known that if Y is multivariate normal then $Y_2|Y_1$ has the form of a linear regression of Y_2 on Y_1, in a Bayesian setting the equivalence of these two models depends on very special properties of the priors used, and in particular of the inverse Wishart distribution (e.g. Geiger and Heckerman (2002), Theorem 5).

Applying Proposition S.4.1 recursively we can consider partitions $Y = (Y_1, Y_2, Y_3)$ into three groups of d_1, d_2 and d_3 variables. This yields

$$Y_1 \sim \text{BMVR}(X; K, \Psi_{11}, m - d_2 - d_3) \tag{S.4.5}$$
$$Y_2|Y_1 \sim \text{BMVR}([X, Y_1]; \tilde{K}, \Psi_{22}, m - d_3) \tag{S.4.6}$$
$$Y_3|Y_1, Y_2 \sim \text{BMVR}([X, Y_1, Y_2]; \tilde{\tilde{K}}, \Psi_{33}, m) \tag{S.4.7}$$

where

$$\tilde{\tilde{K}} = \begin{pmatrix} K & 0 & 0 \\ 0 & \Psi_{11} & 0 \\ 0 & 0 & \Psi_{22} \end{pmatrix}. \tag{S.4.8}$$

Applying this with $X = \mathbf{1}$ and $K = K_0$ gives expressions for the distributions $p(Y_1|\boldsymbol{g}), p(Y_2|Y_1, \boldsymbol{g})$ and $p(Y_3|Y_1, Y_2, \boldsymbol{g})$ under H_0; applying it with $X = [\mathbf{1}, \boldsymbol{g}]$ and $K = K_1$ gives expressions for the same distributions under H_{all}.

To prove Proposition S.4.1 we make use of the following result.

Result S.4.1 (Properties of the Inverse Wishart distribution)**.** *The following properties of the inverse Wishart distribution are well known; see for example Geiger and Heckerman (2002).*

Assume $V \sim W^{-1}(\Psi, m)$, and partition the matrices V and Ψ conformably with

$$V = \begin{pmatrix} V_{11} & V_{12} \\ V_{21} & V_{22} \end{pmatrix} \qquad \Psi = \begin{pmatrix} \Psi_{11} & \Psi_{12} \\ \Psi_{21} & \Psi_{22} \end{pmatrix} \tag{S.4.9}$$

where V_{ij} and Ψ_{ij} are $d_i \times d_j$ matrices. Then

i) V_{11} is independent of $V_{11}^{-1}V_{12}$ and $V_{22.1}$ where $V_{22.1} = V_{22} - V_{21}V_{11}^{-1}V_{12}$.

ii) $V_{11} \sim W^{-1}(\Psi_{11}, m - d_2)$.

iii) $V_{22.1} \sim W^{-1}(\Psi_{22.1}, m)$.

iv) $V_{11}^{-1}V_{12}|V_{22.1} \sim MN_{d_1\times d_2}(\Psi_{11}^{-1}\Psi_{12}, \Psi_{11}^{-1}, V_{22.1})$

Proof of Proposition S.4.1. The multivariate regression model (13) can be written in terms of the matrix normal distribution:

$$Y|B,V \sim \mathrm{MN}_{n\times d}(XB, I_n, V). \tag{S.4.10}$$

Now partition $Y = (Y_1, Y_2), B = (B_1, B_2)$ and

$$V = \begin{pmatrix} V_{11} & V_{12} \\ V_{21} & V_{22} \end{pmatrix}, \tag{S.4.11}$$

where Y_i is $n \times d_i$, B_i is $p \times d_i$, and V_{ij} is $d_i \times d_j$ $(i, j = 1, 2)$. Then, from elementary properties of the multivariate normal distribution, we have

$$Y_1|B,V \sim \mathrm{MN}_{n\times d_1}(XB_1, I_n, V_{11}) \tag{S.4.12}$$
$$Y_2|Y_1,B,V \sim \mathrm{MN}_{n\times d_2}(XB_2 + (Y_1 - XB_1)V_{11}^{-1}V_{12}, I_n, V_{22.1}) \tag{S.4.13}$$
$$\sim \mathrm{MN}_{n\times d_2}\left([X, Y_1]\begin{pmatrix} \tilde{B}_2 \\ R \end{pmatrix}, I_n, V_{22.1}\right) \tag{S.4.14}$$

where $\tilde{B}_2(p \times d_2) = (B_2 - B_1V_{11}^{-1}V_{12})$ and $R(d_1 \times d_2) = V_{11}^{-1}V_{12}$. Note that this is the standard result that says that if Y is multivariate normal, then Y_2 given Y_1 has the form of a linear regression on Y_1.

Now let

$$B|V \sim \mathrm{MN}_{p\times d}(0, K^{-1}, V). \tag{S.4.15}$$

Then again, by elementary properties of the multivariate normal,

$$B_1|V \sim \mathrm{MN}_{p\times d_1}(0, K^{-1}, V_{11}) \tag{S.4.16}$$
$$B_2|B_1,V \sim \mathrm{MN}_{p\times d_2}(B_1V_{11}^{-1}V_{12}, K^{-1}, V_{22.1}). \tag{S.4.17}$$

Note that combining (S.4.12) with (S.4.16) and Property ii), gives (S.4.2). Further, the conditional distribution for B_2, (S.4.19), implies

$$\tilde{B}_2|B_1,V \sim \mathrm{MN}_{p\times d_2}(0, K^{-1}, V_{22.1}). \tag{S.4.18}$$

so in particular $\tilde{B}_2$ is independent of B_1 and R, and combining this with property (iv) above for V we have:

$$\begin{pmatrix} \tilde{B}_2 \\ R \end{pmatrix}|V_{22.1} \sim \mathrm{MN}_{p+d_1\times d_2}(0, \tilde{K}^{-1}, V_{22.1}), \tag{S.4.19}$$

where

$$\tilde{K} = \begin{pmatrix} K & 0 \\ 0 & \Psi_{11} \end{pmatrix}. \tag{S.4.20}$$

Combining (S.4.14) with (S.4.19) and Property iii) gives (S.4.3). □

References

Geiger, D. and D. Heckerman (2002). Parameter priors for directed acyclic graphical models and the characterization of several probability distributions. *Annals of statistics 30*(5), 1412–1440.

Johnson, V. (2008). Properties of bayes factors based on test statistics. *Scandinavian Journal of Statistics 35*(2), 354–368.

Wakefield, J. (2009, Jan). Bayes factors for genome-wide association studies: comparison with p-values. *Genet Epidemiol 33*(1), 79–86.
